# Models and partition of variance for quantitative trait loci with epistasis and linkage disequilibrium

**DOI:** 10.1186/1471-2156-7-9

**Published:** 2006-02-10

**Authors:** Tao Wang, Zhao-Bang Zeng

**Affiliations:** 1 Bioinformatics Research Center & Department of Statistics, North Carolina State University, Raleigh, NC 27695, USA; 2 Department of Genetics, North Carolina State University, Raleigh, NC 27695, USA; 3 Division of Biostatistics & Human Molecular Genetics Center, Medical College of Wisconsin, Milwaukee, Wl 53226, USA

## Abstract

**Background:**

A genetic model about quantitative trait loci (QTL) provides a basis to interpret the genetic basis of quantitative traits in a study population, such as additive, dominance and epistatic effects of QTL and the partition of genetic variance. The standard quantitative genetics model is based on the least squares partition of genetic effects and also genetic variance in an equilibrium population. However, over years many specialized QTL models have also been proposed for applications in some specific populations. How are these models related? How to analyze and partition a QTL model and genetic variance when both epistasis and linkage disequilibrium are considered?

**Results:**

Starting from the classical description of Cockerham genetic model, we first represent the model in a multiple regression setting by using indicator variables to describe the segregation of QTL alleles. In this setting, the definition of additive, dominance and epistatic effects of QTL and the basis for the partition of genetic variance are elaborated. We then build the connection between this general genetic model and a few specialized models (a haploid model, a diploid *F*_2 _model and a general two-allele model), and derive the genetic effects and partition of genetic variance for multiple QTL with epistasis and linkage disequilibrium for these specialized models.

**Conclusion:**

In this paper, we study extensively the composition and property of the genetic model parameters, such as genetic effects and partition of genetic variance, when both epistasis and linkage disequilibrium are considered. This is the first time that both epistasis and linkage disequilibrium are considered in modeling multiple QTL. This analysis would help us to understand the structure of genetic parameters and relationship of various genetic quantities, such as allelic frequencies and linkage disequilibrium, on the definition of genetic effects, and will also help us to understand and properly interpret estimates of the genetic effects and variance components in a QTL mapping experiment.

## Background

Modeling quantitative trait loci (QTL) started with Yule [1,2] and Pearson [3] (see [4,5] for the early history of quantitative genetics). However, it was Fisher [6] who laid the firm foundation for quantitative genetics. Fisher defined gene effects (additive, dominance and epistatic effects) based on the partition of genetic variance. He partitioned the genetic variance into a portion due to additive effects (averaged allelic substitution effects), a portion due to dominance effects (allelic interactions), and a portion due to epistatic effects (non-allelic interactions) of genes. He then studied the correlation between relatives using the model. Cockerham [7] used the orthogonal contrasts to redefine the additive and dominance effects of QTL and, by extending the contrasts to include epistatic effects, he partitioned the epistatic variance of two loci into those due to additive × additive, additive × dominance, dominance × additive and dominance × dominance effects of QTL. Cockerham then generalized the model to multiple loci. This was further generalized by Kempthorne [8,9] to multiple alleles. This model has been used as the basis for studying quantitative genetics ever since.

However, over years, many specialized models have also been proposed. Some are just special cases of the general genetic model and some are simplified variants tailored for particular applications or interpretations. With the propagation of numerous quantitative genetic models, there have also been some confusions in literature on the definition and interpretation of additive, dominance and epistatic effects of QTL and their relationship to the partition of genetic variance. Also, there has never been a study that considers both epistasis and linkage disequilibrium in the partition of genetic variance for multiple QTL. In this paper, we try to build the connection between the general genetic model and a few other commonly used genetic models to clarify the basis for the interpretation of different genetic models.

We start with an introduction of the genetic model as expressed in [7] in the context of variance components. Then by introducing an indicator variable for each QTL allele, we represent the model in a multiple regression setting and examine the definition and meaning of the genetic effects (additive, dominance and epistatic effects) of QTL and partition of the genetic variance in an equilibrium population and also in a disequilibrium population. Most of previous studies on modeling QTL discuss epistasis only in reference to an equilibrium population. An examination of properties of a model with both epistasis and linkage disequilibrium is important for QTL analysis in both experimental and natural populations. This is another goal of the paper and is studied in great detail here. We discuss a few reduced models used for QTL analysis, such as backcross model (essentially a haploid model) and *F*_2 _model. We also give details for a general two-allele model which may be useful for studying the genetic architecture in a natural population using single nucleotide polymorphisms (SNPs).

Previously, in [10], we compared *F*_2 _model and the general two-allele model with another commonly used genetic model, called *F*_∞ _model. By specifying the basis of definition for each model, we compared the properties of these models in the estimation and interpretation of QTL effects including epistasis and discussed a few potential problems of using *F*_∞ _model in a segregating population for QTL analysis. Similarly, we also compared these models with another model proposed by Cheverud [11,12].

An important result of [10] is that the genetic effects defined in reference to an equilibrium population also apply to a disequilibrium population. The partial regression coefficients, that define the genetic effects in a disequilibrium population, equal to the simple regression coefficients in a corresponding equilibrium population – the usual basis to define and interpret a genetic effect including an epistatic effect. Hardy-Weinberg and linkage disequilibria only introduce covariances between different genetic effects. With this result, in this paper our discussion on epistasis and linkage disequilibrium is focused on the partition and composition of genetic variances and covariances between different genetic effects in different populations.

## Results

### The genetic model

A general genetic model for the partition of genetic variance (particularly epistatic variance) in a random mating population was first given by Cockerham [7,13] and extended to multiple alleles by Kempthorne [8,9], following the basic genetic model formulated by Fisher [6]. The model for two loci **A **and **B **with multiple alleles was expressed as follows

$\begin{array}{c} G_{jl}^{ik}=\mu +\alpha^{i}+\alpha_{j}+\delta_{j}^{i}+\beta^{k}+\beta_{l}+\gamma_{l}^{k}+(\alpha^{i}\beta^{k})\\ +(\alpha^{i}\beta_{l})+(\alpha_{j}\beta^{k})+(\alpha_{j}\beta_{l})+(\alpha^{i}\gamma_{l}^{k})\\ +(\alpha_{j}\gamma_{l}^{k})+(\delta_{j}^{i}\beta^{k})+(\delta_{j}^{i}\beta_{l})+(\delta_{j}^{i}\gamma_{l}^{k}) \end{array}\;\left(1\right)$

where the genotypic value $G_{jl}^{ik}$ is the expected phenotype of an individual carrying alleles *A*_*i*_, *A*_*j*_, *B*_*k*_, and *B*_*l *_with phased genotype *A*_*i*_*B*_*k*_/*A*_*j*_*B*_*l *_formed by the union of a paternal gamete *A*_*i*_*B*_*k *_and a maternal gamete *A*_*j*_*B*_*l*_. The model partitions the total genotypic value into a number of genetic effects which include additive effects of each allele (*α*'s and *β*'s), dominance effects between two alleles at each locus (*δ*'s and *γ*'s), additive × additive interactions between two alleles at two loci ((*αβ*)'s), additive × dominance interactions involving three alleles ((*αγ*)'s and (*δβ*)'s), and dominance × dominance interaction involving all four alleles ((*δγ*)'s).

As an ANOVA model, it is known that not all the parameters in model (1) are estimable. A number of constraint conditions on these parameters are therefore needed. Let *p*^*i*^, *q*^*k *^denote allelic frequencies for alleles on paternal gametes, and *p*_*j*_, *q*_*l *_allelic frequencies for alleles on maternal gametes. It is usually assumed that a weighted summation of genetic effects is zero over any index for each genetic component as a deviation from the mean. Some examples are

$\begin{array}{l} \sum_{i}p^{i}\alpha^{i}=0,\;\sum_{i}p^{i}\delta_{j}^{i}=0,\;\sum_{j}p_{j}\delta_{j}^{i}=0,\\ \sum_{i}p^{i}(\alpha^{i}\beta^{k})=0,\;\sum_{k}p^{k}(\alpha^{i}\beta^{k})=0,\\ \sum_{j}p_{j}(\delta_{j}^{i}\beta^{k})=0,\;\sum_{k}q^{k}(\delta_{j}^{i}\beta^{k})=0,\;\cdots \cdots \end{array}\;\left(2\right)$

Under the assumption of *random mating and linkage equilibrium *and allowing for different allelic frequencies in paternal and maternal gametes, the mean and genetic effects can be expressed as follows based on the least squares principle:

$\begin{array}{l} \mu =\sum_{i,j,k,l}p^{i}p_{j}q^{k}q_{l}G_{jl}^{ik}\;\left(3\right)\\ \alpha^{i}=G_{..}^{i.}-G_{..}^{..},\;\beta^{k}=G_{..}^{.k}-G_{..}^{..},\\ \alpha_{j}=G_{j.}^{..}-G_{..}^{..},\;\beta_{l}=G_{.l}^{..}-G_{..}^{..},\\ \delta_{j}^{i}=G_{j.}^{i.}-G_{..}^{i.}-G_{j.}^{..}+G_{..}^{..},\\ \gamma_{l}^{k}=G_{.l}^{.k}-G_{..}^{.k}-G_{.l}^{..}+G_{..}^{..},\\ (\alpha^{i}\beta^{k})=G_{..}^{ik}-G_{..}^{i.}-G_{..}^{.k}+G_{..}^{..},\\ (\alpha^{i}\beta_{l})=G_{.l}^{i.}-G_{..}^{i.}-G_{.l}^{..}+G_{..}^{..},\\ (\alpha_{j}\beta^{k})=G_{j.}^{.k}-G_{j.}^{..}-G_{..}^{.k}+G_{..}^{..},\\ (\alpha_{j}\beta_{l})=G_{jl}^{..}-G_{j.}^{..}-G_{.l}^{..}+G_{..}^{..},\\ (\alpha^{i}\gamma_{l}^{k})=G_{.l}^{ik}-G_{..}^{ik}-G_{.l}^{i.}-G_{.l}^{.k}+G_{..}^{i.}+G_{..}^{.k}\\ +G_{.l}^{..}-G_{..}^{..},\\ (\alpha_{j}\gamma_{l}^{k})=G_{jl}^{.k}-G_{j.}^{.k}-G_{.l}^{.k}-G_{jl}^{..}+G_{j.}^{..}+G_{..}^{.k}\\ +G_{.l}^{..}-G_{..}^{..},\\ (\delta_{j}^{i}\beta^{k})=G_{j.}^{ik}-G_{..}^{ik}-G_{j.}^{i.}-G_{j.}^{.k}+G_{..}^{i.}+G_{..}^{.k}\\ +G_{j.}^{..}-G_{..}^{..},\\ (\delta_{j}^{i}\beta_{l})=G_{jl}^{i.}-G_{j.}^{i.}-G_{.l}^{i.}-G_{jl}^{..}+G_{..}^{i.}+G_{j.}^{..}\\ +G_{.l}^{..}-G_{..}^{..},\\ (\delta_{j}^{i}\gamma_{l}^{k})=G_{jl}^{ik}-G_{j.}^{ik}-G_{.l}^{ik}-G_{jl}^{i.}+G_{jl}^{.k}+G_{..}^{ik}\\ +G_{jl}^{..}+G_{j.}^{i.}+G_{.l}^{.k}+G_{j.}^{.k}+G_{.l}^{i.}-G_{..}^{i.}\\ -G_{..}^{.k}-G_{j.}^{..}-G_{.j}^{..}+G_{..}^{..} \end{array}$

where $G_{..}^{..}=\sum_{i,j,k,l}p^{i}p_{j}q^{k}q_{l}G_{jl}^{ik}$, $G_{..}^{i.}=\sum_{j,k,l}p_{j}q^{k}q_{l}G_{jl}^{ik}$, and so on. The total genetic variance is $V_{G}=\sum_{i,j,k,l}p^{i}p_{j}p^{k}p_{l}{\left(G_{jl}^{ik}-\mu \right)}^{2}$, and has an orthogonal partition under random mating and linkage equilibrium

$\begin{array}{l} V_{G}=V_{A_{1}}+V_{A_{2}}+V_{D_{1}}+V_{D_{2}}+V_{A_{1}A_{2}}+V_{A_{1}D_{2}}\;\left(4\right)\\ +V_{D_{1}A_{2}}+V_{D_{1}D_{2}} \end{array}$

with

$\begin{array}{l} V_{A_{1}}=\sum_{i}p^{i}{\left(\alpha^{i}\right)}^{2}+\sum_{j}p_{j}{\left(\alpha_{j}\right)}^{2}\\ V_{D_{1}}=\sum_{i,j}p^{i}p_{j}{\left(\delta_{j}^{i}\right)}^{2}\\ V_{A_{2}}=\sum_{k}q^{k}{\left(\beta^{k}\right)}^{2}+\sum_{l}q_{l}{\left(\beta_{l}\right)}^{2}\\ V_{D_{2}}=\sum_{k,l}q^{k}q_{l}{\left(\gamma_{l}^{k}\right)}^{2}\\ V_{A_{1}A_{2}}=\sum_{i,k}p^{i}q^{k}{\left(\alpha^{i}\beta^{k}\right)}^{2}+\sum_{j,l}p_{j}q_{l}{\left(\alpha_{j}\beta_{l}\right)}^{2}\\ +\sum_{i,l}p^{i}q_{l}{\left(\alpha^{i}\beta_{l}\right)}^{2}+\sum_{j,k}p_{j}q^{k}{\left(\alpha_{j}\beta^{k}\right)}^{2}\\ V_{A_{1}D_{2}}=\sum_{i,k,l}p^{i}q^{k}q_{l}{\left(\alpha^{i}\gamma_{l}^{k}\right)}^{2}+\sum_{j,k,l}p_{j}q^{k}q_{l}{\left(\alpha_{j}\gamma_{l}^{k}\right)}^{2}\\ V_{D_{1}A_{2}}=\sum_{i,j,k}p^{i}p_{j}q^{k}{\left(\delta_{j}^{i}\beta^{k}\right)}^{2}+\sum_{i,j,l}p^{i}p_{j}q_{l}{\left(\delta_{j}^{i}\beta_{l}\right)}^{2}\\ V_{D_{1}D_{2}}=\sum_{i,j,k,l}p^{i}p_{j}q^{k}q_{l}{\left(\delta_{j}^{i}\gamma_{l}^{k}\right)}^{2} \end{array}$

Using indicator variables, we can represent model (1) in another form. Assume that the two loci **A **and **B **have alleles *A*_*i*_, *i *= 1, 2, ..., *n*_1_; and *B*_*k*_, *i *= 1, 2, ..., *n*_2_, respectively. We define the following indicator variables to represent the segregation of alleles in a population.

$\begin{array}{l} z_{M_{i}}^{\left(1\right)}=\{\begin{array}{ll} 1, & \text{for }A_{i}\text{ allele from paternal gamete}\\ 0, & \text{otherwise}. \end{array}\\ z_{F_{j}}^{\left(1\right)}=\{\begin{array}{ll} 1, & \text{for }A_{j}\text{ allele from maternal gamete}\\ 0, & \text{otherwise}. \end{array} \end{array}$

for *i*, *j *= 1, 2, ..., *n*_1 _at locus **A**, and

$\begin{array}{l} z_{M_{k}}^{\left(2\right)}=\{\begin{array}{ll} 1, & \text{for }B_{k}\text{ allele from paternal gamete}\\ 0, & \text{otherwise}. \end{array}\\ z_{F_{l}}^{\left(2\right)}=\{\begin{array}{ll} 1, & \text{for }B_{l}\text{ allele from maternal gamete}\\ 0, & \text{otherwise}. \end{array} \end{array}$

for *k*, *l *= 1, 2, ..., *n*_2 _at locus **B**. In terms of these indicator variables, we have the following.

• Hardy-Weinberg equilibrium (HWE) implies that {$z_{M_{i}}^{\left(1\right)}$, *i *= 1, 2, ..., *n*_1_} are independent of {$z_{F_{j}}^{\left(1\right)}$, *j *= 1, 2, ..., *n*_1_}, and {$z_{M_{k}}^{\left(2\right)}$, *k *= 1, 2, ..., *n*_2_} are independent of {$z_{F_{l}}^{\left(2\right)}$, *l *= 1, 2, ..., *n*_2_}.

• Linkage equilibrium (LE) implies that {$z_{M_{i}}^{\left(1\right)}$, *i *= 1, 2, ..., *n*_1_} are independent of {$z_{M_{k}}^{\left(2\right)}$, *k *= 1, 2, ..., *n*_2_}, and {$z_{F_{j}}^{\left(1\right)}$, *j *= 1, 2, ..., *n*_1_} are independent of {$z_{F_{l}}^{\left(2\right)}$, *l *= 1, 2, ..., *n*_2_}.

• There is another type of disequilibrium; i.e., the so-called genotypic disequilibrium [14] for two alleles on different gametes and at different loci. So, the genotypic equilibrium (GE) here means that {$z_{M_{i}}^{\left(1\right)}$, *i *= 1, 2,..., *n*_1_} are independent of {$z_{F_{l}}^{\left(2\right)}$, *l *= 1, 2, ..., *n*_2_}, and {$z_{F_{j}}^{\left(1\right)}$, *j *= 1, 2, ..., *n*_1_} are independent of {$z_{M_{k}}^{\left(2\right)}$, *k *= 1, 2, ..., *n*_2_}.

It is known that under random mating we have both HWE and GE, which together are called gametic phase equilibrium. Now, let *G *denote the genotypic value of a progeny drawn randomly from the current population. Based on Cockerham model, *G *can be expressed as

$\begin{array}{l} G=\mu +\sum_{i=1}^{n_{1}}\alpha^{i}z_{M_{i}}^{\left(1\right)}+\sum_{j=1}^{n_{1}}\alpha_{j}z_{F_{j}}^{\left(1\right)}+\sum_{i,j}\delta_{j}^{i}z_{M_{i}}^{\left(1\right)}z_{F_{j}}^{\left(1\right)}\\ +\sum_{k=1}^{n_{2}}\beta^{k}z_{M_{k}}^{\left(2\right)}+\sum_{l=1}^{n_{2}}\beta_{l}z_{F_{l}}^{\left(2\right)}+\sum_{k,l}\gamma_{l}^{k}z_{M_{k}}^{\left(2\right)}z_{F_{l}}^{\left(2\right)}\\ +[\sum_{i,k}(\alpha^{i}\beta^{k})z_{M_{i}}^{\left(1\right)}z_{M_{k}}^{\left(2\right)}+\sum_{i,l}(\alpha^{i}\beta_{l})z_{M_{i}}^{\left(1\right)}z_{F_{l}}^{\left(2\right)}\\ +\sum_{j,k}(\alpha_{j}\beta^{k})z_{F_{j}}^{\left(1\right)}z_{M_{k}}^{\left(2\right)}+\sum_{j,l}(\alpha_{j}\beta_{l})z_{F_{j}}^{\left(1\right)}z_{F_{l}}^{\left(2\right)}]\\ +[\sum_{i,k,l}(\alpha^{i}\gamma_{l}^{k})z_{M_{i}}^{\left(1\right)}z_{M_{k}}^{\left(2\right)}z_{F_{l}}^{\left(2\right)}+\sum_{j,k,l}(\alpha_{j}\gamma_{l}^{k})z_{F_{j}}^{\left(1\right)}z_{M_{k}}^{\left(2\right)}z_{F_{l}}^{\left(2\right)}]\\ +[\sum_{i,j,k}(\delta_{j}^{i}\beta^{k})z_{M_{i}}^{\left(1\right)}z_{F_{j}}^{\left(1\right)}z_{M_{k}}^{\left(2\right)}+\sum_{i,j,l}(\delta_{j}^{i}\beta_{l})z_{M_{i}}^{\left(1\right)}z_{F_{j}}^{\left(1\right)}z_{F_{l}}^{\left(2\right)}]\\ +\sum_{i,j,k,l}(\delta_{j}^{i}\gamma_{l}^{k})z_{M_{i}}^{\left(1\right)}z_{F_{j}}^{\left(1\right)}z_{M_{k}}^{\left(2\right)}z_{F_{l}}^{\left(2\right)}\;\left(5\right) \end{array}$

This is simply a different presentation of Cockerham model with the same constraint conditions applied on the coefficient parameters. For a given individual with genotype *A*_*i*_*B*_*k*_/*A*_*j*_*B*_*l*_, *G *will take the same value of $G_{jl}^{ik}$ as before. However, this expression is helpful for us to understand some details about each component of genetic effects. We can see this more clearly in the examination of some reduced models later.

In general, the genetic effects can be defined separately for alleles that are paternally and maternally transmitted to account for possible biological differences. As a fully parameterized model for $G_{jl}^{ik}$, model (3) may give $G_{jl}^{ik}\neq G_{jk}^{il}\neq G_{il}^{jk}\neq G_{ik}^{jl}$ depending on how genetic effects are defined. If locus **A **has *n*_1 _alleles, and locus **B **has *n*_2 _alleles, there are *N *= $n_{1}^{2}n_{2}^{2}$ possible phased genotypes in total with the partition of the degrees of freedom given in Table 1.

**Table 1:** Partition of degrees of freedom for two loci with number of alleles *n*_1 _and *n*_2 _(a general case)

Source	Degrees of Freedom
additive (*α*)	2(*n*_1 _- 1)
dominance (*δ*)	(*n*_1 _- 1)^2^
additive (*β*)	2(*n*_2 _- 1)
dominance (*γ*)	(*n*_2 _- 1)^2^
additive × additive (*αβ*)	4(*n*_1 _- 1)(*n*_2 _- 1)
additive × dominance (*αγ*)	2(*n*_1 _- 1)(*n*_2 _- 1)^2^
dominance × additive (*δβ*)	2(*n*_1 _- 1)^2^(*n*_2 _- 1)
dominance × dominance (*δγ*)	(*n*_1 _- 1)^2^(*n*_2 _- 1)^2^

total	$n_{1}^{2}n_{2}^{2}$ - 1

If we assume that the union of paternal gamete *A*_*i*_*B*_*k *_with maternal gamete *A*_*j*_*B*_*l *_have the same mean effect as that of paternal gamete *A*_*j*_*B*_*l *_with maternal gamete *A*_*i*_*B*_*k*_(i.e., $G_{jl}^{ik}=G_{ik}^{jl}$), the coupling and repulsion heterozygotes have the same genotypic value (i.e., $G_{jl}^{ik}=G_{jk}^{il}$), and paternal and maternal gametes have the same gametic frequency distribution, we do not need to distinguish paternal and maternal effects. In this case, the two loci can be regarded as 2 factors and each factor has $n_{i}^{2}$ (*i *= 1, 2) levels produced by the allelic combinations of *n*_*i *_alleles (cf. [7]). The total number of genotypes is *N *= *n*_1_(*n*_1 _+ 1)*n*_2_(*n*_2 _+ 1)/4 and the partition of degrees of freedom is shown in Table 2. Since in this case, *α*^*i *^= *α*_*i*_, *β*^*k *^= *β*_*k*_, ..., and so on, the model can also be expressed as follows

$\begin{array}{l} G=\mu +\sum_{i=1}^{n_{1}}\alpha_{i}(z_{M_{i}}^{\left(1\right)}+z_{F_{i}}^{\left(1\right)})+\sum_{i,j}\delta_{j}^{i}z_{M_{i}}^{\left(1\right)}z_{F_{j}}^{\left(1\right)}\\ +\sum_{k=1}^{n_{2}}\beta_{k}(z_{M_{k}}^{\left(2\right)}+z_{F_{k}}^{\left(2\right)})+\sum_{k,l}\gamma_{l}^{k}z_{M_{k}}^{\left(2\right)}z_{F_{l}}^{\left(2\right)}\\ +\sum_{i,k}\left(\alpha_{i}\beta_{k}\right)(z_{M_{i}}^{\left(1\right)}+z_{F_{i}}^{\left(1\right)})(z_{M_{k}}^{\left(2\right)}+z_{F_{k}}^{\left(2\right)})\\ +\sum_{i,k,l}\left(\alpha_{i}\gamma_{l}^{k}\right)(z_{M_{i}}^{\left(1\right)}+z_{F_{i}}^{\left(1\right)})z_{M_{k}}^{\left(2\right)}z_{F_{l}}^{\left(2\right)}\\ +\sum_{i,j,k}\left(\delta_{j}^{i}\beta_{k}\right)z_{M_{i}}^{\left(1\right)}z_{F_{j}}^{\left(1\right)}(z_{M_{k}}^{\left(2\right)}+z_{F_{k}}^{\left(2\right)})\\ +\sum_{i,j,k,l}\left(\delta_{j}^{i}\gamma_{l}^{k}\right)z_{M_{i}}^{\left(1\right)}z_{F_{j}}^{\left(1\right)}z_{M_{k}}^{\left(2\right)}z_{F_{l}}^{\left(2\right)})\;\left(6\right) \end{array}$

**Table 2:** Partition of degrees of freedom for two loci with number of alleles *n*_1 _and *n*_2 _(a simplified case without distinguishing the paternal and maternal origins)

Source	Degrees of Freedom
additive (*α*)	*n*_1 _- 1
dominance (*δ*)	*n*_1_(*n*_1 _- 1)/2
additive (*β*)	*n*_2 _- 1
dominance (*γ*)	*n*_2_(*n*_2 _- 1)/2
additive × additive (*αβ*)	(*n*_1 _- 1)(*n*_2 _- 1)
additive × dominance (*αγ*)	(*n*_1 _- 1)*n*_2_(*n*_2 _- 1)/2
dominance × additive (*δβ*)	*n*_1_(*n*_1 _- 1)(*n*_2 _- 1)/2
dominance × dominance (*δγ*)	*n*_1_(*n*_1 _- 1)*n*_2_(*n*_2 _- l)/4

total	$\frac{n_{1}(n_{1}+1)}{2}\cdot \frac{n_{2}(n_{2}+1)}{2}-1$

For the case of an arbitrary number of loci, the situation will become more complicated. In addition to the additive and dominance effects at each locus and two locus interactions (additive × additive, additive × dominance, dominance × additive, dominance × dominance, with a total number of 2^2 ^terms), there are 3 locus interactions (additive × additive × additive, additive × additive × dominance, ..., with a total number of 2^3 ^terms), 4 locus interactions (additive × additive × additive × additive, ..., with a total number of 2^4 ^terms), and so on. Though the extension is straightforward, the total number of terms will increase dramatically. We will show some models with multiple loci in later examples by ignoring trigenic and higher order epistasis.

### Effects and variance components

Let *p*^*i*^, *p*_*j *_(*i*, *j *= 1, 2, ..., *n*_1_) be allelic frequencies of paternal and maternal gametes at locus **A**, respectively. Let also *q*^*k*^, *q*_*l *_(*k*, *l *= 1, 2, ..., *n*_2_) denote allelic frequencies of paternal and maternal gametes at locus **B**, respectively. In the analysis of variance for the model, it is convenient to use deviations of the indicator variables $z_{M_{i}}^{\left(1\right)}$, $z_{F_{i}}^{\left(1\right)}$, $z_{M_{j}}^{\left(2\right)}$ and $z_{F_{j}}^{\left(2\right)}$ from their expected values. That is

$\begin{array}{l} x_{M_{i}}^{\left(1\right)}=z_{M_{i}}^{\left(1\right)}-E(z_{M_{i}}^{\left(1\right)})=z_{M_{i}}^{\left(1\right)}-p^{i}\\ =\{\begin{array}{ll} 1-p^{i}, & \text{for }A_{i}\text{ allele from paternal gamete}\\ -p^{i}, & \text{otherwise} \end{array} \end{array}$

Similarly, define

$\begin{array}{l} x_{F_{j}}^{\left(1\right)}=z_{F_{j}}^{\left(1\right)}-E(z_{F_{j}}^{\left(1\right)})=z_{F_{j}}^{\left(1\right)}-p_{j}\\ x_{M_{k}}^{\left(2\right)}=z_{M_{k}}^{\left(2\right)}-E(z_{M_{k}}^{\left(2\right)})=z_{M_{k}}^{\left(2\right)}-q^{k}\\ x_{F_{l}}^{\left(2\right)}=z_{F_{l}}^{\left(2\right)}-E(z_{F_{l}}^{\left(2\right)})=z_{F_{l}}^{\left(2\right)}-q_{l} \end{array}$

Taking the constraint conditions on the genetic effects into account, we can show that,

$\begin{array}{c} \sum_{i=1}^{n_{1}}\alpha^{i}x_{M_{i}}^{\left(1\right)}=\sum_{i=1}^{n_{1}}\alpha^{i}z_{M_{i}}^{\left(1\right)}\\ \sum_{i,j}\delta_{j}^{i}x_{M_{i}}^{\left(1\right)}x_{F_{j}}^{\left(1\right)}=\sum_{i,j}\delta_{j}^{i}z_{M_{i}}^{\left(1\right)}z_{F_{j}}^{\left(1\right)}\\ \sum_{i,k,l}(\alpha^{i}\gamma_{l}^{k})x_{M_{i}}^{\left(1\right)}x_{M_{k}}^{\left(2\right)}x_{F_{l}}^{\left(2\right)}=\sum_{i,k,l}(\alpha^{i}\gamma_{l}^{k})z_{M_{i}}^{\left(1\right)}z_{M_{k}}^{\left(2\right)}z_{F_{l}}^{\left(2\right)}\\ \sum_{i,j,k,l}(\delta_{j}^{i}\gamma_{l}^{k})x_{M_{i}}^{\left(1\right)}x_{F_{j}}^{\left(1\right)}x_{M_{k}}^{\left(2\right)}x_{F_{l}}^{\left(2\right)}=\sum_{i,j,k,l}(\delta_{j}^{i}\gamma_{l}^{k})z_{M_{i}}^{\left(1\right)}z_{F_{j}}^{\left(1\right)}z_{M_{k}}^{\left(2\right)}z_{F_{l}}^{\left(2\right)} \end{array}$

and so on. For example,

$\begin{array}{l} \sum_{i=1}^{n_{1}}\alpha^{i}x_{M_{i}}^{\left(1\right)}=\sum_{i=1}^{n_{1}}\alpha^{i}(z_{M_{i}}^{\left(1\right)}-p^{i})\\ =\sum_{i=1}^{n_{1}}\alpha^{i}z_{M_{i}}^{\left(1\right)}-\sum_{i=1}^{n_{1}}\alpha^{i}p^{i}=\sum_{i=1}^{n_{1}}\alpha^{i}z_{M_{i}}^{\left(1\right)} \end{array}$

as $\sum_{i=1}^{n_{1}}\alpha^{i}p^{i}=0$ by the constrain condition (2). Therefore, model (5) can be rewritten as

$\begin{array}{l} G=\mu +\sum_{i=1}^{n_{1}}\alpha^{i}x_{M_{i}}^{\left(1\right)}+\sum_{j=1}^{n_{1}}\alpha^{i}x_{F_{j}}^{\left(1\right)}+\sum_{i,j}\delta_{j}^{i}x_{M_{i}}^{\left(1\right)}x_{F_{j}}^{\left(1\right)}\\ +\sum_{k=1}^{n_{2}}\beta^{k}x_{M_{k}}^{\left(2\right)}+\sum_{l=1}^{n_{2}}\beta_{l}x_{F_{l}}^{\left(2\right)}+\sum_{k,l}\gamma_{l}^{k}x_{M_{k}}^{\left(2\right)}x_{F_{l}}^{\left(2\right)}\\ +[\sum_{i,k}(\alpha^{i}\beta^{k})x_{M_{i}}^{\left(1\right)}x_{M_{k}}^{\left(2\right)}+\sum_{i,l}(\alpha^{i}\beta_{l})x_{M_{i}}^{\left(1\right)}x_{F_{l}}^{\left(2\right)}\\ +\sum_{j,k}(\alpha_{j}\beta^{k})x_{F_{j}}^{\left(1\right)}x_{M_{k}}^{\left(2\right)}+\sum_{j,l}\left(\alpha_{j}\beta_{l})x_{F_{j}}^{\left(1\right)}x_{F_{l}}^{\left(2\right)}\right]\\ +[\sum_{i,k,l}(\alpha^{i}\gamma_{l}^{k})x_{M_{i}}^{\left(1\right)}x_{M_{k}}^{\left(2\right)}x_{F_{l}}^{\left(2\right)}+\sum_{j,k,l}(\alpha_{j}\gamma_{l}^{k})x_{F_{j}}^{\left(1\right)}x_{M_{k}}^{\left(2\right)}x_{F_{l}}^{\left(2\right)}]\\ +[\sum_{i,j,k}(\delta_{j}^{i}\beta^{k})x_{M_{i}}^{\left(1\right)}x_{F_{j}}^{\left(1\right)}x_{M_{k}}^{\left(2\right)}+\sum_{i,j,l}(\delta_{j}^{i}\beta_{l})x_{M_{i}}^{\left(1\right)}x_{F_{j}}^{\left(1\right)}x_{F_{l}}^{\left(2\right)}]\\ +\sum_{i,j,k,l}(\delta_{j}^{i}\gamma_{l}^{k})x_{M_{i}}^{\left(1\right)}x_{F_{j}}^{\left(1\right)}x_{M_{k}}^{\left(2\right)}x_{F_{l}}^{\left(2\right)}\;\left(7\right) \end{array}$

When we deal with some reduced models in the next section, we will find that the model of this form is especially helpful as it makes the model parameter constraints built into regression variables which is suited for genetic interpretation. The form of model (7) can also facilitate the demonstration that under Hardy-Weinberg, linkage and genotypic equilibria, the regression coefficients (genetic effects) are Cockerham's least squares effects (3) (Appendix A), and the genotypic variance *V*_*G *_has the orthogonal partition (4) (Appendix B).

Now we discuss the properties of model in a disequilibrium situation. As stated in [14], there are three types of disequilibria

• Typel: between alleles on the same gametes but at different loci

• Type2: between alleles at the same locus but on different gametes

• Type3: between alleles on different gametes and at different loci.

If we denote $P_{jl}^{ik}$ as the genotypic frequency of *A*_*i*_*B*_*k*_/*A*_*j*_*B*_*l*_, $P_{j.}^{i.}$ as the genotypic frequency of *A*_*i*_/*A*_*j*_, and so on, following [14], the digenic disequilibria can be written as

$\begin{array}{l} D_{..}^{ik}=\text{Cov}(z_{M_{i}}^{\left(1\right)},z_{M_{k}}^{\left(2\right)})=E(x_{M_{i}}^{\left(1\right)}x_{M_{k}}^{\left(2\right)})=P_{..}^{ik}-p^{i}q^{k}\\ D_{jl}^{..}=\text{Cov}(z_{F_{j}}^{\left(1\right)},z_{F_{l}}^{\left(2\right)})=E(x_{F_{j}}^{\left(1\right)}x_{F_{l}}^{\left(2\right)})=P_{jl}^{..}-p_{j}q_{l}\\ D_{j.}^{i.}=E(x_{M_{i}}^{\left(1\right)}x_{F_{j}}^{\left(1\right)})=P_{j.}^{i.}-p^{i}p_{j}\\ D_{.l}^{.k}=E(x_{M_{k}}^{\left(2\right)}x_{F_{l}}^{\left(2\right)})=P_{.l}^{.k}-q^{k}q_{l}\\ D_{.j}^{i.}=E(x_{M_{i}}^{\left(1\right)}x_{F_{l}}^{\left(2\right)})=P_{.l}^{i.}-p^{i}q_{l}\\ D_{j.}^{.k}=E(x_{F_{j}}^{\left(1\right)}x_{M_{k}}^{\left(2\right)})=P_{j.}^{.k}-p_{j}q^{k}. \end{array}$

And the trigenic disequilibria

$\begin{array}{c} D_{j.}^{ik}=E(x_{M_{i}}^{\left(1\right)}x_{F_{j}}^{\left(1\right)}x_{M_{k}}^{\left(2\right)})\\ =P_{j.}^{ik}-p^{i}P_{j.}^{.k}-q^{k}P_{j.}^{i.}-p_{j}P_{..}^{ik}+2p^{i}p_{j}q^{k}\\ D_{.l}^{ik}=E(x_{M_{i}}^{\left(1\right)}x_{M_{k}}^{\left(2\right)}x_{F_{l}}^{\left(2\right)})\\ =P_{.l}^{ik}-p^{i}P_{.l}^{.k}-q^{k}P_{.l}^{i.}-q_{l}P_{..}^{ik}+2p^{i}q^{k}q_{l}\\ D_{jl}^{i.}=E(x_{M_{i}}^{\left(1\right)}x_{F_{j}}^{\left(1\right)}x_{F_{l}}^{\left(2\right)})\\ =P_{jl}^{i.}-p^{i}P_{jl}^{..}-p_{j}P_{..}^{i.}-q_{l}P_{j.}^{i.}+2p^{i}p_{j}q_{l}\\ D_{jl}^{.k}=E(x_{F_{j}}^{\left(1\right)}x_{M_{k}}^{\left(2\right)}x_{F_{l}}^{\left(2\right)})\\ =P_{jl}^{.k}-p_{j}P_{.l}^{.k}-q^{k}P_{j.}^{.l}-q_{l}P_{j.}^{.k}+2p_{j}q^{k}q_{l} \end{array}$

Similarly for the quadrigenic disequilibrium, we may define

$\begin{array}{c} D_{jl}^{ik}=E(x_{M_{i}}^{\left(1\right)}x_{F_{j}}^{\left(1\right)}x_{M_{k}}^{\left(2\right)}x_{F_{l}}^{\left(2\right)})\\ =P_{jl}^{ik}-p^{i}P_{jl}^{.k}-p_{j}P_{.l}^{ik}-q^{k}P_{jl}^{i.}-q_{l}P_{j.}^{ik}\\ +p^{i}p_{j}P_{.l}^{.k}-p^{i}q^{k}P_{jl}^{..}+p^{i}q_{l}P_{j.}^{.k}+p_{j}q^{k}P_{.l}^{i.}\\ +p_{j}q_{l}P_{..}^{ik}+q^{k}q_{l}P_{j.}^{i.}-3p^{i}p_{j}q^{k}q_{l}. \end{array}$

If we express $D_{jl}^{ik}$ as a function of lower-order linkage disequilibria, we have

$\begin{array}{c} D_{jl}^{ik}=P_{jl}^{ik}-p^{i}D_{jl}^{.k}-p_{j}D_{.l}^{ik}-q^{k}D_{jl}^{i.}-q_{l}D_{j.}^{ik}\\ -p^{i}p_{j}D_{.l}^{.k}-p^{i}q^{k}D_{jl}^{..}-p^{i}q_{l}D_{j.}^{.k}-p_{j}q^{k}D_{.l}^{i.}\\ -p_{j}q_{l}D_{..}^{ik}-q^{k}q_{l}D_{j.}^{i.}-p^{i}p_{j}q^{k}q_{l}. \end{array}$

This definition is the same as that given by [15,16]. Note that $\sum_{i}z_{M_{i}}^{\left(1\right)}=1$. Then, we have

$\begin{array}{l} \sum_{i}D_{jl}^{ik}=E[(\sum_{i}x_{M_{i}}^{\left(1\right)})x_{F_{j}}^{\left(1\right)}x_{M_{k}}^{\left(2\right)}x_{F_{l}}^{\left(2\right)}]\\ =E[\sum_{i}(z_{M_{i}}^{\left(1\right)}-p^{i})x_{F_{j}}^{\left(1\right)}x_{M_{k}}^{\left(2\right)}x_{F_{l}}^{\left(2\right)}=0 \end{array}$

In general, $D_{jl}^{ik}$ is summed to zero over any allele involved, so are $D_{j.}^{ik}$ and other disequilibrium measurements.

With Hardy-Weinberg and genotypic equilibria but linkage disequilibrium, model (7) leads to the following expression for the overall mean

$\begin{array}{c} E(G)=\mu +\sum_{i,k}(\alpha^{i}\beta^{k})D_{..}^{ik}+\sum_{j,l}(\alpha_{j}\beta_{l})D_{jl}^{..}\\ +\sum_{i,j,k,l}(\delta_{j}^{i}\gamma_{l}^{k})D^{ik}D_{jl} \end{array}$

where *μ *is the mean genotypic value under linkage equilibrium, and $\Delta_{\mu }=\sum_{i,k}\left(\alpha^{i}\beta^{k}\right)D_{..}^{ik}+\sum_{j,l}\left(\alpha_{j}\beta_{l}\right)D_{jl}^{..}+\sum_{i,j,k,l}\left(\delta_{j}^{i}\gamma_{l}^{k}\right)D_{..}^{ik}D_{jl}^{..}$ represents the departure from *μ *due to linkage disequilibrium and epistasis. If there is no epistasis, linkage disequilibrium does not affect the mean genotypic value. Similar results were given by [17]. Note that for marginal means of the genotypic values, we have

$\begin{array}{l} G_{..}^{..}=\sum_{i,j,k,l}P_{..}^{ik}P_{jl}^{..}G_{jl}^{ik},\;G_{..}^{i.}=\frac{1}{p^{i}}\sum_{j,k,l}P_{..}^{ik}P_{jl}^{..}G_{jl}^{ik},\\ G_{..}^{ik}=\sum_{j,l}P_{jl}^{..}G_{jl}^{ik},\;G_{j.}^{i.}=\frac{1}{p^{i}p_{j}}\sum_{k,l}P_{jl}^{..}P_{..}^{ik}G_{jl}^{ik},\\ G_{j.}^{ik}=\frac{1}{p_{j}}\sum_{l}P_{jl}^{..}G_{jl}^{ik},\;... \end{array}$

and so on.

Then the question is what the genetic effects are in a disequilibrium population. Do Hardy-Weinberg and linkage disequilibria change the definition and values of genetic effects? The short answer to this question is "no" in a fully characterized model, but "yes" in a model that ignores some QTL or genetic effects. This is proved and discussed in [10]. With Hardy-Weinberg and linkage disequilibria, the genetic effects no longer correspond to the deviations from marginal means of genotypic values in a disequilibrium population. In a multiple regression model (7), the genetic effects are partial regression coefficients. These partial regression coefficients correspond to the simple regression coefficients, or deviations from marginal means of genotypic values, only in an equilibrium population. In a disequilibrium population, a direct analysis on the partial regression coefficients can be very complex (see the appendix of [10] for a relatively simple example). However, in a full model which includes all relevant loci and genetic effects, the model parameters depend only on how the regressors, i.e. *x *variables in (7), are defined and are independent of correlations between *x *variables, i.e. Hardy-Weinberg and linkage disequilibria. So, the genetic effects are still the same as those defined in the equilibrium population, although the population mean and marginal means of genotypic values are changed in a disequilibrium population.

Hardy-Weinberg and linkage disequilibria introduce correlation between *x *variables, thus covariances between different genetic effect components. Define

$\begin{array}{l} A_{1}=\sum_{i=1}^{n_{1}}\alpha^{i}x_{M_{i}}^{\left(1\right)}+\sum_{j=1}^{n_{1}}\alpha_{j}x_{F_{j}}^{\left(1\right)}\\ D_{1}=\sum_{i,j}\delta_{j}^{i}x_{M_{i}}^{\left(1\right)}x_{F_{j}}^{\left(1\right)}\\ A_{2}=\sum_{k=1}^{n_{2}}\beta^{k}x_{M_{k}}^{\left(2\right)}+\sum_{l=1}^{n_{2}}\beta_{l}x_{F_{l}}^{\left(2\right)}\\ D_{2}=\sum_{k,l}\gamma_{l}^{k}x_{M_{k}}^{\left(2\right)}x_{F_{l}}^{\left(2\right)}\\ A_{1}A_{2}=\sum_{i,k}(\alpha^{i}\beta^{k})x_{M_{i}}^{\left(1\right)}x_{M_{k}}^{\left(2\right)}+\sum_{i,l}(\alpha^{i}\beta_{l})x_{M_{i}}^{\left(1\right)}x_{F_{l}}^{\left(2\right)}\\ +\sum_{j,k}(\alpha_{j}\beta^{k})x_{F_{j}}^{\left(1\right)}x_{M_{k}}^{\left(2\right)}+\sum_{j,l}(\alpha_{j}\beta_{l})x_{F_{j}}^{\left(1\right)}x_{F_{l}}^{\left(2\right)}\\ A_{1}D_{2}=\sum_{i,k,l}(\alpha^{i}\gamma_{l}^{k})x_{M_{i}}^{\left(1\right)}x_{M_{k}}^{\left(2\right)}x_{F_{l}}^{\left(2\right)}\\ +\sum_{j,k,l}(\alpha_{j}\gamma_{l}^{k})x_{F_{j}}^{\left(1\right)}x_{M_{k}}^{\left(2\right)}x_{F_{l}}^{\left(2\right)}\\ A_{2}D_{1}=\sum_{i,j,k}(\delta_{j}^{i}\beta^{k})x_{M_{i}}^{\left(1\right)}x_{F_{j}}^{\left(1\right)}x_{M_{k}}^{\left(2\right)}\\ +\sum_{i,j,l}(\delta_{j}^{i}\beta_{l})x_{M_{i}}^{\left(1\right)}x_{F_{j}}^{\left(1\right)}x_{F_{l}}^{\left(2\right)}\\ D_{1}D_{2}=\sum_{i,j,k,l}(\delta_{j}^{i}\gamma_{l}^{k})x_{M_{i}}^{\left(1\right)}x_{F_{j}}^{\left(1\right)}x_{M_{k}}^{\left(2\right)}x_{F_{l}}^{\left(2\right)} \end{array}$

Then we can write

*G *= *μ *+ *A*_1 _+ *A*_2 _+ *D*_1 _+ *D*_2 _+ *A*_1_*A*_2 _+ *A*_1_*D*_2 _+ *A*_2_*D*_1 _+ *D*_1_*D*_2_

In a disequilibrium population, the partition of the genotypic variance becomes

$V_{G}=\sum_{i=1}^{8}\sum_{j=1}^{8}V_{ij}=1^{T}V1$

where

*V *= (*V*_*ij*_)_8 × 8_

It is a symmetric matrix. In Appendix C, we give the detailed result for each component of the matrix with linkage disequilibrium, but assuming Hardy-Weinberg equilibrium.

For the rest of paper, when we discuss disequilibrium, we mainly discuss linkage disequilibrium and assume Hardy-Weinberg and genotypic equilibria which can be achieved by random mating in one generation. Hardy-Weinberg disequilibrium can be taken into account which will make results more complex and is thus omitted.

### Reduced models

In many genetic applications, experimental population has some regular genetic structure by design. In these cases, the genetic model can be further simplified to reflect the experimental design structure. Also sometimes we may want to simplify the genetic model by imposing certain constrains or assumptions, such as the number of alleles, to increase the feasibility of analysis. In this section, we give a few reduced genetic models that are relevant to many genetic applications.

#### 1. Backcross population or recombinant inbred population (haploid model)

Backcross population or recombinant inbred population is a common experimental design for QTL mapping study. By crossing two inbred lines, we can create a *F*_1 _population. If we randomly backcross *F*_1 _to one of the inbred lines, we have a backcross population. Let us assume that the cross is *AA *(paternal) × *Aa *(maternal). In a random-mating backcross population, there are only two possible genotypes at each segregating locus *A*_*r*_*A*_*r *_or *A*_*r*_*a*_*r*_, for *r *= 1, 2, ..., *m*, where *m *is the number of QTL. Since for the paternal gametes,

$z_{M_{1}}^{\left(r\right)}=\{\begin{array}{ll} 1, & \text{for }A_{r}\text{ allele from paternal gamete}\\ 0, & \text{otherwise } \end{array}=1,$

and

$z_{M_{2}}^{\left(r\right)}=\{\begin{array}{ll} 1, & \text{for }a_{r}\text{ allele from paternal gamete}\\ 0, & \text{otherwise } \end{array}=0,$

thus $x_{M_{1}}^{\left(r\right)}=z_{M_{1}}^{\left(r\right)}-1=0$ and $x_{M_{2}}^{\left(r\right)}=z_{M_{2}}^{\left(r\right)}=0$ for *r *= 1, 2, ..., *m*. For maternal gametes however,

$\begin{array}{c} x_{F_{1}}^{\left(r\right)}=\{\begin{array}{l} 1/2,\text{ for }A_{r}\text{ from maternal gamete}\\ -1/2,\text{ otherwise} \end{array}\\ =-x_{F_{2}}^{\left(r\right)},\text{ for }r=1,2,\cdot \cdot \cdot ,m. \end{array}$

Thus the model becomes

$\begin{array}{c} G=\mu +\sum_{r=1}^{m}a_{r}x_{F_{1}}^{\left(r\right)}+\sum_{r<s}b_{rs}(x_{F_{1}}^{\left(r\right)}x_{F_{1}}^{\left(s\right)})\\ +\sum_{r<s<t}c_{rst}(x_{F_{1}}^{\left(r\right)}x_{F_{1}}^{\left(s\right)}x_{F_{1}}^{\left(t\right)})+\cdots \end{array}\;\left(8\right)$

where $a_{r}=\alpha_{1}^{\left(r\right)}-\alpha_{2}^{\left(r\right)}$ is the substitution effect between homozygote genotype *A*_*r*_*A*_*r *_and heterozygote genotype $A_{r}a_{r},b_{rs}=(\alpha_{1}^{\left(r\right)}\alpha_{1}^{\left(s\right)})-(\alpha_{1}^{\left(r\right)}\alpha_{2}^{\left(s\right)})-(\alpha_{2}^{\left(r\right)}\alpha_{1}^{\left(s\right)})+(\alpha_{2}^{\left(r\right)}\alpha_{2}^{\left(s\right)})$ is the interaction effect between loci *r *and $s,c_{rst}=(\alpha_{1}^{\left(r\right)}\alpha_{1}^{\left(s\right)}\alpha_{1}^{\left(t\right)})-(\alpha_{1}^{\left(r\right)}\alpha_{1}^{\left(s\right)}\alpha_{2}^{\left(t\right)})-(\alpha_{1}^{\left(r\right)}\alpha_{2}^{\left(s\right)}\alpha_{1}^{\left(t\right)})+(\alpha_{1}^{\left(r\right)}\alpha_{2}^{\left(s\right)}\alpha_{2}^{\left(t\right)})-(\alpha_{2}^{\left(r\right)}\alpha_{1}^{\left(s\right)}\alpha_{1}^{\left(t\right)})+(\alpha_{2}^{\left(r\right)}\alpha_{1}^{\left(s\right)}\alpha_{2}^{\left(t\right)})+(\alpha_{2}^{\left(r\right)}\alpha_{2}^{\left(s\right)}\alpha_{1}^{\left(t\right)})-(\alpha_{2}^{\left(r\right)}\alpha_{2}^{\left(s\right)}\alpha_{2}^{\left(t\right)})$, ..., and so on. Taking constraint conditions into account, we have *α*_1 _= -*α*_2_, *β*_1 _= -*β*_2_, and so on. Then, *a*_*r *_= $2\alpha_{1}^{\left(r\right)}$, *b*_*rs *_= 4($\alpha_{1}^{\left(r\right)}\alpha_{1}^{\left(s\right)}$), and *c*_*rst *_= 8($\alpha_{1}^{\left(r\right)}\alpha_{1}^{\left(s\right)}\alpha_{1}^{\left(t\right)}$), and so on. With linkage equilibrium, the genetic effects as the partial regression coefficients of the model correspond to the simple regression coefficients. For example, for the substitution effect of locus *r*, *a*_*r*_, it is the covariance between genotypic value *G *and substitution effect design variable $x_{F_{1}}^{\left(r\right)}$ divided by the variance of $x_{F_{1}}^{\left(r\right)}$. So in general, we have

$\begin{array}{c} a_{r}=E[(G-\mu )x_{F_{1}}^{\left(r\right)}]/E(x_{F_{1}}^{(r)2})\\ =E[G(z_{F_{1}}^{\left(r\right)}-1/2)]/(1/4)\\ =2[E(G|z_{F_{1}}^{\left(r\right)}=1)-E(G)]\\ b_{rs}=E[(G-\mu )x_{F_{1}}^{\left(r\right)}x_{F_{1}}^{\left(s\right)}]/[E(x_{F_{1}}^{(r)2})E(x_{F_{1}}^{(s)2})]\\ \begin{array}{l} =4[E(G|z_{F_{1}}^{\left(r\right)}=z_{F_{1}}^{\left(s\right)}=1)-E(G|z_{F_{1}}^{\left(r\right)}=1)\\ -E(G|z_{F_{1}}^{\left(s\right)}=1)+E(G)] \end{array}\\ c_{rst}=E[(G-\mu )x_{F_{1}}^{\left(r\right)}x_{F_{1}}^{\left(s\right)}x_{F_{1}}^{\left(t\right)}]/\\ {}[E(x_{F_{1}}^{(r)2})E(x_{F_{1}}^{(s)2})E(x_{F_{1}}^{(t)2})]\\ \begin{array}{l} =8[E(G|z_{F_{1}}^{\left(r\right)}=z_{F_{1}}^{\left(s\right)}=z_{F_{1}}^{\left(t\right)}=1)\\ -E(G|z_{F_{1}}^{\left(r\right)}=z_{F_{1}}^{\left(s\right)}=1)\\ -E(G|z_{F_{1}}^{\left(r\right)}=z_{F_{1}}^{\left(t\right)}=1)\\ -E(G|z_{F_{1}}^{\left(s\right)}=z_{F_{1}}^{\left(t\right)}=1)\\ +E(G|z_{F_{1}}^{\left(r\right)}=1)+E(G|z_{F_{1}}^{\left(s\right)}=1)\\ +E(G|z_{F_{1}}^{\left(t\right)}=1)-E(G)] \end{array} \end{array}$

The orthogonal partition of the genotypic variance in an equilibrium population is

$V_{G}=\frac{1}{4}\sum_{r=1}^{l}a_{r}^{2}+\frac{1}{4^{2}}\sum_{r<s}b_{rs}^{2}+\frac{1}{4^{3}}\sum_{r<s<t}c_{rst}^{2}+\cdots \;\left(9\right)$

As noted above, linkage disequilibrium does not change the values of genetic effects in a full model. The model parameters are still the same as those defined in the equilibrium population. However, in this case there is a simple relationship between the substitution effects at multiple loci and marginal means of genotypic values in a disequilibrium population [18]. This is noted here. Let $P_{rs}=P\{z_{F_{1}}^{\left(r\right)}=z_{F_{1}}^{\left(s\right)}=1\}$, and the digenic linkage disequilibrium be defined as

$D_{rs}=\text{Cov(}z_{F_{1}}^{\left(r\right)},z_{F_{1}}^{\left(s\right)})=E(x_{F_{1}}^{\left(r\right)}x_{F_{1}}^{\left(s\right)})$

Ignoring trigenic and higher order linkage disequilibria, we have

$E\left[(G-\mu )x_{F_{1}}^{\left(r\right)}\right]=\frac{1}{4}a_{r}+\sum_{r'\neq r}D_{rr'}a_{r'}$

$E[(G-\mu )x_{F_{1}}^{\left(r\right)}x_{F_{1}}^{\left(s\right)}]=\frac{1}{4^{2}}b_{rs}$

Therefore, the digenic interaction effects can be expressed as

$\begin{array}{l} b_{rs}=4^{2}[P_{rs}E(G|z_{F_{1}}^{\left(r\right)}=z_{F_{1}}^{\left(s\right)}=1)-D_{rs}\mu ]\\ -4[E(G|z_{F_{1}}^{\left(r\right)}=1)+E(G|z_{F_{1}}^{\left(s\right)}=1)-E(G)] \end{array}$

Then the substitution effects can be expressed as a function of marginal means in the disequilibrium population as

$\left(\begin{array}{c} a_{1}\\ a_{2}\\ \vdots \\ a_{l} \end{array}\right)={\left(I+4D\right)}^{-1}(2q)$

where *I *is a *m *× *m *identity matrix, *D *= (*D*_*ij*_)_*m *× *m *_with all diagonal elements being zeros;

**q **= (*q*_1_, *q*_2_, ..., *q*_*m*_,)^*T*^, with *q*_*i *_= *E*(*G*|$z_{F_{1}}^{\left(i\right)}$ = 1) - *E*(*G*), for *i *= 1, 2, ..., *m*.

The partition of genetic variance with linkage disequilibrium is complex. Here we give details of the partition of genotypic variance for the following model

$G=\mu +\sum_{r=1}^{m}a_{r}x_{F_{1}}^{\left(r\right)}+\sum_{r<s}b_{rs}(x_{F_{1}}^{\left(r\right)}x_{F_{1}}^{\left(s\right)})\;\left(10\right)$

Let *x*_*r *_= $x_{F_{1}}^{\left(r\right)}$ and *x*_*s *_= $x_{F_{1}}^{\left(s\right)}$ to simplify the notation here. The genotypic variance is

$\begin{array}{l} V_{G}=V(\sum_{r}a_{r}x_{r})+2\text{Cov(}\sum_{r}a_{r}x_{r},\sum_{r<s}b_{rs}x_{r}x_{s})\\ +\text{V}(\sum_{r<s}b_{rs}x_{r}x_{s})\\ =\sum_{r}a_{r}^{2}p_{r}(1-p_{r})+2\sum_{r<s}a_{r}a_{s}D_{rs}\\ +2\sum_{r<s}\left[a_{r}b_{rs}(1-2p_{r})D_{rs}+a_{s}b_{rs}(1-2p_{s})D_{rs}\right]\\ +2\sum_{r<s<t}(a_{r}b_{st}+a_{s}b_{rt}+a_{t}b_{rs})D_{rst}\\ +\sum_{r<s}b_{rs}^{2}[(1-2p_{r})(1-2p_{s})D_{rs}\\ +p_{r}(1-p_{r})p_{s}(1-p_{s})-D_{rs}^{2}]\\ +2\sum_{r<s<t}\{b_{rs}b_{rt}[(1-2p_{r})D_{rst}\\ -p_{r}(1-p_{r})D_{st}-D_{rs}D_{rt}]\\ +b_{rs}b_{st}[(1-2p_{s})D_{rst}+p_{s}(1-p_{s})D_{rt}-D_{rs}D_{st}]\\ +b_{rt}b_{st}[(1-2p_{t})D_{rst}\\ +p_{t}(1-p_{t})D_{rs}-D_{rt}D_{st}]\}\\ +2\sum_{r<s<t<u}[b_{rt}b_{su}(D_{rstu}-D_{rt}D_{su})\\ +b_{ru}b_{st}(D_{rstu}-D_{ru}D_{st})\\ +b_{rs}b_{tu}(D_{rstu}-D_{rs}D_{tu})]\;\left(11\right) \end{array}$

where

*D*_*rst *_= *E*(*x*_*r*_*x*_*s*_*x*_*t*_) and *D*_*rstu *_= *E*(*x*_*r*_*x*_*s*_*x*_*t*_*x*_*u*_)

are three locus and four locus linkage disequilibria. This is a general partition of genetic variance for a haploid model.

For the backcross population, it can be shown that *D*_*rst *_= 0 (see Appendix D for both backcross and *F*_2 _populations) and *D*_*rstu *_= *D*_*rs*_*D*_*tu *_for loci *r*, *s*, *t *and *u *in this order under the assumption of no crossing-over interference. Also with this assumption, *D*_*rt *_= 4*D*_*rs*_*D*_*st *_and *D*_*rs *_= (1 - 2*λ*_*rs*_)/4, where *λ*_*rs *_is the recombination frequency between loci *r *and *s*. Since, *p*_*r *_= *p*_*s *_= 1/2, the variance becomes

$\begin{array}{lll} V_{G}= & \frac{1}{4}\sum_{r}a_{r}^{2}+2\sum_{r<s}a_{r}a_{s}D_{rs}+ & \\ & \frac{1}{16}\sum_{r<s}b_{rs}^{2}(1-16D_{rs}^{2}) & \\ & +\frac{1}{2}\sum_{r<s<t}[b_{rs}b_{rt}(1-16D_{rs}^{2})D_{st} & \\ & +b_{rt}b_{st}(1-16D_{st}^{2})D_{rs}] & \\ & +2\sum_{r<s<t<u}\left(b_{rt}b_{su}+b_{ru}b_{st}\right) & \\ & (1-16D_{st}^{2})D_{rs}D_{tu} & \;\left(12\right) \end{array}$

In this partition of variance, the first summation term is the genetic variance due to the substitution effect of each QTL, the second summation term is the covariance between substitution effects of QTL pairs due to linkage disequilibrium, the third summation term is the genetic variance due to epistatic effects of QTL, and the fourth and fifth summation terms are the covariance between different epistatic effects of QTL due to linkage disequilibrium. There is no covariance between the main substitution effects and epistatic effects (see also [19]).

For a backcross population, the genetic interpretation of the substitution effect *a*_*r *_depends on which parental line is backcrossed. In one backcross *AA *× *Aa*, the substitution effect is traditionally defined as the difference between the additive effect and dominance effects, and in the other backcross *Aa *× *aa*, it is the sum of the additive and dominance effects. Only with both backcrosses, can one estimate both additive and dominance effects separately (for example [20]).

The same model also applies to a recombinant inbred population which is another very popular experimental design for QTL mapping study. For a recombinant inbred population, the substitution effects of QTL are the additive effects and the epistatic effects are the additive × additive interaction effects. Statistical methods to map QTL and to estimate various components of the genetic variance due to QTL including epistasis has been developed through the maximum likelihood approach [19,21]. In a few cases where the method was applied, we estimated, for the first time, how the quantitative genetic variance was partitioned into various components in designed experimental populations. For example, Weber *et al. *[22] reported the result of QTL mapping for wing shape on the third chromosome of *Drosophila melanogaster *from a cross of divergent selection lines. From 519 recombinant inbred lines, 11 QTL were mapped on the third chromosome. Nine QTL pairs showed significant epistatic effects. The total genetic variance amounts to 95.5% of the phenotypic variance in the recombinant inbred lines with phenotypes measured and averaged over 50 male flies for each recombinant inbred line. The partition of the genetic variance is as follows (see Table 6 and 7 of [22]): 27.4% due to the variances of additive effects (equivalent to the first summation term of (12)); 67.3% due to the covariances between additive effects (the second summation term); 7.2% due to the variances of epistatic effects (the third summation term); and -6.0% due to the covariances between epistatic effects (the fourth and fifth summation terms). The covariances between additive and epistatic effects, expected to be 0, account for -0.4% due to sampling. Similar kind of partition of the genetic variance is also observed in a group of 701 second chromosome recombinant inbred lines from a cross of the same divergent selection lines (see Table 4 and 5 of [23]). See also [20] for another example.

#### 2. *F*_2 _population

*F*_2 _is created from a cross between pairs of *F*_1 _individuals. It is also a very popular experimental design for QTL mapping study. The advantage of this design is that both additive and dominance effects of a QTL can be estimated as well as various epistatic effects. The design also has more statistical power for QTL detection as compared to a backcross population. In a random-mating *F*_2 _population, there are only two alleles at each segregating locus and allelic frequencies are expected to be one half if there is no segregation distortion.

Let us consider only two loci first. Let *A *and *a *denote the two alleles at locus 1, and *B *and *b *at locus 2. In this case, $x_{M_{1}}^{\left(1\right)}=-x_{M_{2}}^{\left(1\right)}$ and $x_{F_{1}}^{\left(1\right)}=-x_{F_{2}}^{\left(1\right)}$. Assuming $G_{jl}^{ij}=G_{ik}^{jl}=G_{jk}^{il}=G_{il}^{jk}$, it also holds that *α*^1 ^= *α*_1 _= -*α*^2 ^= -*α*_2_, $\delta_{1}^{1}=\delta_{2}^{2}=-\delta_{1}^{2}$, and so on. The additive term for locus 1 then becomes

$A_{1}=\alpha^{1}(x_{M_{1}}^{\left(1\right)}-x_{M_{2}}^{\left(1\right)})+\alpha_{1}(x_{F_{1}}^{\left(1\right)}-x_{F_{2}}^{\left(1\right)})=2\alpha_{1}\;w_{1}$

with

$w_{1}=x_{M_{1}}^{\left(1\right)}+x_{F_{1}}^{\left(1\right)}=\{\begin{array}{l} 1,\text{ for }AA\text{ at locus 1}\\ \text{0, for }Aa\text{ at locus 1}\\ -1,\text{ for }aa\text{ at locus 1} \end{array}\;\left(13\right)$

and the dominance term is

$D_{1}=2(\delta_{1}^{1})(x_{M_{1}}^{\left(1\right)}x_{F_{1}}^{\left(1\right)}+x_{M_{1}}^{\left(1\right)}x_{F_{1}}^{\left(1\right)})=(-2)(\delta_{1}^{1})v_{1}$

with

$v_{1}=(-2)x_{M_{1}}^{\left(1\right)}x_{F_{1}}^{\left(1\right)}=\{\begin{array}{c} -1/2,\text{ for }AA\text{ at locus 1}\\ 1/2,\text{ for }Aa\text{ at locus 1}\\ -1/2,\text{ for }aa\text{ at locus 1} \end{array}\;\left(14\right)$

Note that the *v *variable in this section for *F*_2 _differ, by a factor -2, from the *v *variable in the next section for a general two-allele model to conform to the usual definition for the *F*_2 _model. Similarly, for locus 2

*A*_2 _= 2*β*_1_*w*_2 _and *D*_2 _= (-2)($\gamma_{\text{1}}^{\text{2}}$)*v*_2_

with

$w_{2}=\{\begin{array}{c} 1,\text{ for }BB\text{ at locus 2}\\ 0,\text{ for }Bb\text{ at locus 2}\\ -1,\text{ for }bb\text{ at locus 2} \end{array}$

and

$v_{2}=\{\begin{array}{c} -1/2,\text{ for }BB\text{ at locus 2}\\ 1/2,\text{ for }Bb\text{ at locus 2}\\ -1/2,\text{ for }bb\text{ at locus 2} \end{array}\;\left(15\right)$

The model can then be written as

$\begin{array}{c} G=\mu +a_{1}w_{1}+d_{1}v_{1}+a_{2}w_{2}+d_{2}v_{2}\\ +{\left(aa\right)}_{12}(w_{1}w_{2})+{\left(ad\right)}_{12}(w_{1}v_{2})\\ +{\left(da\right)}_{12}(v_{1}w_{2})+{\left(dd\right)}_{12}(v_{1}v_{2}) \end{array}\;\left(16\right)$

where the parameters are related as *a*_1 _= 2*α*_1_, *a*_2 _= 2*β*_2_, *d*_1 _= - $-2\delta_{1}^{1}$, *d*_2 _= -$-2\gamma_{1}^{1}$, (*aa*)_12 _= 4(*α*_1_*β*_1_), (*ad*)_12 _= 8($\alpha_{1}\gamma_{1}^{1}$), (*da*)_12 _= 8($\delta_{1}^{1}\beta_{1}$), (*dd*)_12 _= 16($\delta_{1}^{1}\gamma_{1}^{1}$). With random mating and linkage equilibrium, we have

$\begin{array}{c} a_{1}=E[G-\mu )w_{1}]/E(w_{1}^{2})=2(G_{1.}^{..}-G_{..}^{..})\\ d_{1}=E[(G-\mu )v_{1}]/E(v_{2}^{1})\\ =(-2)(G_{1.}^{1.}-2G_{1.}^{..}+G_{..}^{..})\\ a_{2}=2(G_{.1}^{..}-G_{..}^{..})\\ d_{2}=(-2)(G_{.1}^{.1}-2G_{.1}^{..}+G_{..}^{..})\\ {\left(aa\right)}_{12}=E[(G-\mu )w_{1}w_{2}]/E(w_{1}^{2}w_{2}^{2})\\ =4(G_{..}^{11}-G_{1.}^{..}-G_{.1}^{..}+G_{..}^{..}\\ {\left(ad\right)}_{12}=E[(G-\mu )w_{1}v_{2}]/E(w_{1}^{2}v_{2}^{2})\\ =(-4)(G_{11}^{.1}-2G_{11}^{..}-G_{.1}^{.1}+G_{1.}^{..}+2G_{.1}^{..}-G_{..}^{..})\\ {\left(da\right)}_{12}=E[(G-\mu )v_{1}w_{2}]/E(v_{1}^{2}w_{2}^{2})\\ =(-4)(G_{11}^{1.}-2G_{11}^{..}-G_{1.}^{1.}+G_{.1}^{..}+2G_{1.}^{..}-G_{..}^{..})\\ {\left(dd\right)}_{12}=E[(G-\mu )v_{1}v_{2}]/E(v_{1}^{2}v_{2}^{2})\\ =4(G_{11}^{11}-2G_{11}^{1.}-2G_{11}^{.1}+G_{1.}^{1.}+G_{.1}^{.1}+4G_{11}^{..}\\ -2G_{1.}^{..}-2G_{.1}^{..}+G_{..}^{..}) \end{array}$

The orthogonal partition of the genotypic variance is

$\begin{array}{cc} V_{G}=\frac{1}{2}a_{1}^{2}+\frac{1}{4}d_{1}^{2}+\frac{1}{2}a_{2}^{2}+\frac{1}{4}d_{2}^{2}+\frac{1}{4}{\left(aa\right)}_{12}^{2} & \\ +\frac{1}{8}{\left(ad\right)}_{12}^{2}+\frac{1}{8}{\left(da\right)}_{12}^{2}+\frac{1}{16}{\left(dd\right)}_{12}^{2}. & \;\left(17\right) \end{array}$

Recently, Kao and Zeng [18] have examined many genetic and statistical issues of the above *F*_2 _model and the effects of linkage disequilibrium. As we have shown here, the *F*_2 _model is a special case of Cockerham model with two alleles at each locus and all allelic frequencies being 1/2.

Now we give the partition of genetic variance for *m *loci with epistasis and linkage disequilibrium in the *F*_2 _population. Generalizing model (16) to *m *loci and ignoring the trigenic and higher order epistasis, we have the following model

$\begin{array}{cc} G=\mu +\sum_{r=1}^{m}a_{r}w_{r}+\sum_{r=1}^{m}d_{r}v_{r}+\sum_{r<s}{\left(aa\right)}_{rs}(w_{r}w_{s}) & \\ +\sum_{r\neq s}{\left(ad\right)}_{rs}(w_{r}v_{s})+\sum_{r<s}{\left(dd\right)}_{rs}(v_{r}v_{s}) & \\ =\mu +A+D+AA+Ad+DD & \;\left(18\right) \end{array}$

The partition of genetic variance for this model under the assumption of Hardy-Weinberg equilibrium is

$\begin{array}{ll} V_{G}=V_{A}+V_{D}+V_{AA}+V_{AD}+V_{DD} & \\ +2\text{Cov}(A,D)+2\text{Cov}(A,AA)+2\text{Cov}(A,AD) & \\ +2\text{Cov(A,DD)+2Cov}(D,AA)+2\text{Cov}(D,AD) & \\ +2\text{Cov(}D,DD)+2Cov(AA,AD) & \\ +2\text{Cov(}AA,DD)+2\text{Cov}(AD,DD). & \;\left(19\right) \end{array}$

The detail of each component is presented in Appendix D.

The *F*_2 _model is a special case of the general two-allele model with *p*_*r *_= 1/2. Note the difference on the *v *variable used for the *F*_2 _model and for the general two-allele model below. This partition of genetic variance can provide a basis for the interpretation of genetic variance estimation by multiple interval mapping in a *F*_2 _population [19,21].

#### 3. A general two-allele model

Here, we provide details of a general two-allele model for multiple loci. This model is probably useful for studying genetic architecture of a quantitative trait in natural populations. Let the two alleles at locus *r *be *A*_*r *_and *a*_*r *_for *r *= 1, 2, ..., *m *with *m *the number of QTL. Assume that the frequencies and genetic effects of alleles are the same for both paternal and maternal gametes. Let *p*_*r *_denote the frequency of allele *A*_*r *_at locus *r*. Note that in this case $z_{M_{1}}^{\left(r\right)}=1-z_{M_{2}}^{\left(r\right)}$, *r *= 1, 2, ..., *m*. Also $x_{M_{1}}^{\left(r\right)}=z_{M_{1}}^{\left(r\right)}-E[z_{M_{1}}^{\left(r\right)}]=(1-z_{M_{2}}^{\left(r\right)})-E(1-z_{M_{2}}^{\left(r\right)})=-x_{M_{2}}^{\left(r\right)}$. Similarly, $x_{F_{2}}^{\left(r\right)}=-x_{F_{1}}^{\left(r\right)}$ Ignoring higher order epistasis involving at least three loci, we can define a two-allele model as

$\begin{array}{cc} G=\mu +\sum_{r=1}^{m}a_{r}w_{r}+\sum_{r=1}^{m}d_{r}v_{r}+\sum_{r<s}{\left(aa\right)}_{rs}(w_{r}w_{s}) & \\ +\sum_{r\neq s}{\left(ad\right)}_{rs}(w_{r}v_{s})+\sum_{r<s}{\left(dd\right)}_{rs}(v_{r}v_{s}) & \;\left(20\right) \end{array}$

where

$\begin{array}{c} w_{r}=x_{M_{1}}^{\left(r\right)}+x_{F_{1}}^{\left(r\right)}\\ =\{\begin{array}{cc} \left(1-p_{r}\right) & \text{for }A_{r}A_{r}\text{ at locus }r\\ 1-2p_{r} & \text{for }A_{r}a_{r}\text{ at locus }r\\ -2p_{r} & \text{for }a_{r}a_{r}\text{ at locus }r \end{array}\;\left(21\right) \end{array}$

$\begin{array}{c} v_{r}=x_{M_{1}}^{\left(x\right)}x_{F_{1}}^{\left(x\right)}\\ =\{\begin{array}{ll} {\left(1-p_{r}\right)}^{2} & \text{for }A_{r}A_{r}\text{ at locus }r\\ -p_{r}(1-p_{r}) & \text{for }A_{r}a_{r}\text{ at locus }r\\ p_{r}^{2} & \text{for }a_{r}a_{r}\text{ at locus }r \end{array}\;\left(22\right) \end{array}$

for *r *= 1, 2, ..., *m*. The coefficients are associated with the original parameters in Cockerham model as follows.

$\begin{array}{cc} a_{r}= & \alpha_{1}^{\left(r\right)}-\alpha_{2}^{\left(r\right)}\\ d_{r}= & \delta_{1}^{1(r)}-\delta_{2}^{1(r)}-\delta_{1}^{2(r)}+\delta_{2}^{2(r)}\\ {\left(aa\right)}_{rs}= & \left(\alpha_{1}^{\left(r\right)}\alpha_{1}^{\left(s\right)})-(\alpha_{1}^{\left(r\right)}\alpha_{2}^{\left(s\right)})-(\alpha_{2}^{\left(r\right)}\alpha_{1}^{\left(s\right)}\right)\\ & +(\alpha_{2}\alpha_{2}^{\left(s\right)})\\ {\left(ad\right)}_{rs}= & \left(\alpha_{1}^{\left(r\right)}\delta_{1}^{1(s)})-(\alpha_{1}^{\left(r\right)}\delta_{2}^{1(s)})-(\alpha_{1}^{\left(r\right)}\delta_{1}^{2(s)}\right)\\ & +(\alpha_{1}^{\left(r\right)}\delta_{2}^{2(s)})-(\alpha_{2}^{\left(r\right)}\delta_{1}^{1(s)})+(\alpha_{2}^{\left(r\right)}\delta_{2}^{1(s)})\\ & +(\alpha_{2}^{\left(r\right)}\delta_{1}^{2(s)})-(\alpha_{2}^{\left(r\right)}\delta_{2}^{2(s)})\\ {\left(dd\right)}_{rs}= & \left(\delta_{1}^{1(r)}\delta_{1}^{1(s)})-(\delta_{1}^{1(r)}\delta_{2}^{1(s)})-(\delta_{1}^{1(r)}\delta_{1}^{2(s)}\right)\\ & +(\delta_{1}^{1(r)}\delta_{2}^{2(s)})-(\delta_{2}^{1(r)}\delta_{1}^{1(s)})+(\delta_{2}^{1(r)}\delta_{2}^{1(s)})\\ & +(\delta_{2}^{1(r)}\delta_{1}^{2(s)})-(\delta_{2}^{1(r)}\delta_{2}^{2(s)})-(\delta_{1}^{2(r)}\delta_{1}^{1(s)})\\ & +(\delta_{1}^{2(r)}\delta_{2}^{1(s)})+(\delta_{1}^{2(r)}\delta_{1}^{2(s)})-(\delta_{1}^{2(r)}\delta_{2}^{2(s)})\\ & \begin{array}{l} +(\delta_{2}^{2(r)}\delta_{1}^{1(s)})-(\delta_{2}^{2(r)}\delta_{2}^{1(s)})-(\delta_{2}^{2(r)}\delta_{1}^{2(s)})\\ +(\delta_{2}^{2(r)}\delta_{2}^{2(s)}) \end{array} \end{array}$

The constraint conditions further lead to

$\begin{array}{c} a_{r}=\alpha_{1}^{\left(r\right)}-\frac{\left(-p_{r}\right)}{\left(1-p_{r}\right)}\alpha_{1}^{\left(r\right)}=\frac{1}{\left(1-p_{r}\right)}\alpha_{1}^{\left(r\right)}\\ d_{r}=\frac{1}{{\left(1-p_{r}\right)}^{2}}\delta_{1}^{1(r)}\\ {\left(aa\right)}_{rs}=\frac{1}{\left(1-p_{r})(1-p_{s}\right)}(\alpha_{1}^{\left(r\right)}\alpha_{1}^{\left(s\right)})\\ {\left(ad\right)}_{rs}=\frac{1}{(1-p_{r}){\left(1-p_{s}\right)}^{2}}(\alpha_{1}^{\left(r\right)}\delta_{1}^{1(s)})\\ {\left(dd\right)}_{rs}=\frac{1}{{\left(1-p_{r}\right)}^{2}{\left(1-p_{s}\right)}^{2}}(\delta_{1}^{1(r)}\delta_{1}^{1(s)}) \end{array}$

With Hardy-Weinberg, linkage and genotypic equilibria, the partial regression coefficients in the above model correspond to the simple regression coefficients

$\begin{array}{lll} a_{r} & = & \frac{1}{\left(1-p_{r}\right)}[E(G|z_{M_{1}}^{\left(r\right)}=1)-E(G)]\\ d_{r} & = & \frac{1}{{\left(1-p_{r}\right)}^{2}}[E(G|z_{M_{1}}^{\left(r\right)}=z_{F_{1}}^{\left(r\right)}=1)\\ & & -2E(G|z_{M_{1}}^{\left(r\right)}=1)+E(G)]\\ {\left(aa\right)}_{rs} & = & \frac{1}{\left(1-p_{r})(1-p_{s}\right)}[E(G|z_{M_{1}}^{\left(r\right)}=z_{M_{1}}^{\left(s\right)}=1)\\ & & -E(G|z_{M_{1}}^{\left(r\right)}=1)-E(G|z_{M_{1}}^{\left(s\right)}=1)+E(G)]\\ {\left(ad\right)}_{rs} & = & \frac{1}{(1-p_{r}){\left(1-p_{s}\right)}^{2}}[E(G|z_{M_{1}}^{\left(r\right)}=z_{M_{1}}^{\left(s\right)}=z_{F_{1}}^{\left(s\right)}=1)\\ & & -2E(G|z_{M_{1}}^{\left(r\right)}=z_{M_{1}}^{\left(s\right)}=1)\\ & & -E(G|z_{M_{1}}^{\left(s\right)}=z_{F_{1}}^{\left(s\right)}=1)+E(G|z_{M_{1}}^{\left(r\right)}=1)\\ & & +2E(G|z_{M_{1}}^{\left(s\right)}=1)-E(G)]\\ {\left(dd\right)}_{rs} & = & \frac{1}{{\left(1-p_{r}\right)}^{2}{\left(1-p_{s}\right)}^{2}}\\ & & \left[E(G|z_{M_{1}}^{\left(r\right)}=z_{F_{1}}^{\left(r\right)}=z_{M_{1}}^{\left(s\right)}=z_{F_{1}}^{\left(s\right)}=1\right)\\ & & -2E(G|z_{M_{1}}^{\left(r\right)}=z_{F_{1}}^{\left(r\right)}=z_{M_{1}}^{\left(s\right)}=1)\\ & & -2E(G|z_{M_{1}}^{\left(r\right)}=z_{M_{1}}^{\left(s\right)}=z_{F_{1}}^{\left(s\right)}=1)\\ & & -E(G|z_{M_{1}}^{\left(r\right)}=z_{F_{1}}^{\left(r\right)}=1)\\ & & +E(G|z_{M_{1}}^{\left(s\right)}=z_{F_{1}}^{\left(r\right)}=1)\\ & & +4E(G|z_{M_{1}}^{\left(r\right)}=z_{F_{1}}^{\left(s\right)}=1)\\ & & -2E(G|z_{M_{1}}^{\left(r\right)}=1)-2E(G|z_{M_{1}}^{\left(s\right)}=1)\\ & & +E(G)] \end{array}$

Note that in this case the genetic effects in the original model are

$\begin{array}{lll} \alpha_{1}^{\left(r\right)} & = & E(G|z_{M_{1}}^{\left(r\right)}=1)-E(G)\\ \delta_{1}^{1}(r) & = & E(G|z_{M_{1}}^{\left(r\right)}=z_{F_{1}}^{\left(r\right)}=1)\\ & & -2E(G|z_{M_{1}}^{\left(r\right)}=1)+E(G)\\ \left(\alpha_{1}^{\left(r\right)}\alpha_{1}^{\left(s\right)}\right) & = & E(G|z_{M_{1}}^{\left(r\right)}=z_{M_{1}}^{\left(s\right)}=1)\\ & & -E(G|z_{M_{1}}^{\left(r\right)}=1)-E(G|z_{M_{1}}^{\left(s\right)}=1)\\ & & +E(G)\\ \left(\alpha_{1}^{\left(r\right)}\delta_{1}^{1(s)}\right) & = & E(G|z_{M_{1}}^{\left(r\right)}=z_{M_{1}}^{\left(s\right)}=z_{F_{1}}^{\left(s\right)}=1)\\ & & -2E(G|z_{M_{1}}^{\left(r\right)}=z_{M_{1}}^{\left(s\right)}=1)\\ & & -E(G|z_{M_{1}}^{\left(s\right)}=z_{F_{1}}^{\left(s\right)}=1)\\ & & +E(G|z_{M_{1}}^{\left(r\right)}=1)+2E(G|z_{M_{1}}^{\left(s\right)}=1)\\ & & -E(G)\\ \left(\delta_{1}^{1(r)}\delta_{1}^{1(s)}\right) & = & E(G|z_{M_{1}}^{\left(r\right)}=z_{F_{1}}^{\left(r\right)}=z_{M_{1}}^{\left(s\right)}=z_{F_{1}}^{\left(s\right)}=1)\\ & & -2E(G|z_{M_{1}}^{\left(r\right)}=z_{F_{1}}^{\left(r\right)}=z_{M_{1}}^{\left(s\right)}=1)\\ & & -2E(G|z_{M_{1}}^{\left(r\right)}=z_{M_{1}}^{\left(s\right)}=z_{F_{1}}^{\left(s\right)}=1)\\ & & +E(G|z_{M_{1}}^{\left(r\right)}=z_{F_{1}}^{\left(r\right)}=1]\\ & & +E(G|z_{M_{1}}^{\left(s\right)}=z_{F_{1}}^{\left(s\right)}=1)\\ & & +4E(G|z_{M_{1}}^{\left(r\right)}=z_{F_{1}}^{\left(s\right)}=1)\\ & & -2E(G|z_{M_{1}}^{\left(r\right)}=1)-2E(G|z_{M_{1}}^{\left(s\right)}=1)\\ & & +E(G) \end{array}$

They are the same as the least squares definition.

Yet another form of this result is shown in Table 1 of [10]. Zeng et al. [10] also show that linkage disequilibrium does not change the values of genetic effects in a full model. This means that the partial regression coefficients in a disequilibrium population equal to the simple regression coefficients in a corresponding equilibrium population with the same allelic frequency configuration.

The partition of genotypic variance in an equilibrium population is

$\begin{array}{cc} V_{G}=2\sum_{r=1}^{m}p_{r}(1-p_{r})a_{r}^{2}+\sum_{r=1}^{m}p_{r}^{2}{\left(1-p_{r}\right)}^{2}d_{r}^{2} & \\ +4\sum_{r<s}p_{r}p_{s}(1-p_{r})(1-p_{s}){\left(aa\right)}_{rs}^{2} & \\ +2\sum_{r\neq s}p_{r}(1-p_{r})p_{s}^{2}{\left(1-p_{s}\right)}^{2}{\left(ad\right)}_{rs}^{2} & \\ +\sum_{r<s}p_{r}^{2}p_{s}^{2}{\left(1-p_{r}\right)}^{2}{\left(1-p_{s}\right)}^{2}{\left(dd\right)}_{rs}^{2} & \;\left(23\right) \end{array}$

or

$\begin{array}{c} V_{G}=\frac{2p_{r}}{\left(1-p_{r}\right)}\sum_{r=1}^{m}{\left(\alpha_{1}^{\left(r\right)}\right)}^{2}+\frac{p_{r}^{2}}{{\left(1-p_{r}\right)}^{2}}\sum_{r=1}^{m}{\left(\delta_{1}^{1(r)}\right)}^{2}\\ +\frac{4p_{r}p_{s}}{\left(1-p_{r})(1-p_{s}\right)}\sum_{r<s}{\left(\alpha_{1}^{\left(r\right)}\alpha_{1}^{\left(s\right)}\right)}^{2}\\ +\frac{2p_{r}p_{s}^{2}}{(1-p_{r}){\left(1-p_{s}\right)}^{2}}\sum_{r\neq s}{\left(\alpha_{1}^{\left(r\right)}\delta_{1}^{1(s)}\right)}^{2}\\ +\frac{p_{r}^{2}p_{s}^{2}}{{\left(1-p_{r}\right)}^{2}{\left(1-p_{s}\right)}^{2}}\sum_{r<s}{\left(\delta_{1}^{1(r)}\delta_{1}^{1(s)}\right)}^{2} \end{array}\;\left(24\right)$

The partition of the genetic variance with epistasis and linkage disequilibrium is complex. We give the result with trigenic and quadrigenic linkage disequilibria included as well. The partition of variance has a similar form as (19). The detail of each component is presented in Appendix E.

## Discussion

In this paper we explore various properties of the standard quantitative genetic model with multiple interacting loci in linkage equilibrium and disequilibrium. Starting from the traditional least squares model, we represent it in the setting of multiple regression with standardized allelic indicator variables and their products as the independent variables and the trait value as the dependent variable. Then the partial regression coefficients associated with these indicator variables define the additive, dominance and epistatic effects for QTL. This is the original definition of QTL effects introduced by Fisher [6] and extended to epistasis by Cockerham [7]. We examine the properties and meaning of these QTL effects in an equilibrium population and also in a disequilibrium population. We show details of the partition of genetic variance for both equilibrium and disequilibrium populations in terms of QTL effects, allelic frequencies and disequilibrium measures. Moreover, we relate this general model to several reduced models used for QTL mapping analysis in cross populations from inbred lines, such as *F*_2_, backcross and recombinant inbred lines. The detailed partition of genetic variance in these populations can provide a basis for the interpretation of genetic variance component estimates from multiple interval mapping [21].

The purpose of modeling QTL is to provide a meaningful and convenient framework and basis to infer and interpret relative significance of each QTL and intricate inter-relationship among QTL on a set of quantitative traits in an experimental or natural population for genetic study. The linear model provides a framework to study the effects of QTL on the mean and variance of the distribution of a trait or multiple traits in a population. With the assumption of a normal distribution for both genotypic and phenotypic values of a quantitative trait or multiple traits, this analysis on the first and second order statistics is sufficient to characterize the relationship between QTL and trait (s). Otherwise, it is an approximation on the relationship. In this model, the model parameters are partitioned into two parts: one is the effects of QTL (additive, dominance and epistatic effects), and the other is frequencies and correlations (Hardy-Weinberg and linkage disequilibria) of QTL alleles. Together they characterize the genetic architecture of quantitative traits in a population.

This linear model also provides a framework for statistical inference of genetic model parameters. If QTL genotypes are known and directly observed, a regression analysis of trait phenotype on QTL genotypes would provide a direct estimation of the genetic model parameters. However, if QTL genotypes are unknown and are only indirectly observed through molecular markers, the statistical inference of QTL and model parameters becomes more complicated. Statistically, we can regard QTL genotypes as missing data with trait phenotypes and marker genotypes as observed data and use a mixture model through the maximum likelihood analysis to infer the conditional distribution of missing data and through that to infer QTL parameters which also include the number and genomic position of QTL [19,24,25]. The likely positions of QTL are searched in the whole genome if data permit and the number of significant QTL positions can be estimated through some model selection procedure.

On modeling QTL, the consistence of model parameters in a multiple-locus setting is an important consideration. It is important for a model to be multiple-locus consistent, and the relationship within and between loci can be clearly and readily analyzed, estimated and interpreted. Here the consistence means that the effect of a QTL is consistently defined in a reference equilibrium population for one, two or multiple loci. In statistics, this is the property of orthogonality. This property is particularly important for the study of epistasis. With that the additive, dominance and epistatic effects can be independently and consistently estimated for one, two, three or multiple loci in the reference population where the model is defined and interpreted. Thus, if the number of QTL is incorrectly identified which seems to be always the case in practice, the parameter values for those identified QTL can still be consistently estimated. However, the situation would certainly be different and complicated if the population is not at equilibrium, for example for QTL in linkage disequilibrium. Linkage disequilibrium would complicate the partition of genetic variance, and could certainly bias the estimation of parameter values for those identified QTL if the QTL model (number and genomic position of QTL) is miss-identified.

In this paper, we study extensively the composition and property of the genetic model parameters, such as genetic effects and partition of genetic variance, when both epistasis and linkage disequilibrium are considered. This would help us to understand the relationship of various genetic quantities, such as allelic frequencies and linkage disequilibrium, on the definition of genetic effects. It would also help us to understand and properly interpret estimates of the genetic effects and variance components in a QTL mapping experiment. It is important to emphasize that modeling QTL is inherently population based as it defines the variation of QTL in reference to a population, either a study population, cross population or natural population. The very basic concept of additive effect of a QTL is a population concept and is population dependent. It depends on the genotypes at other loci and depends on the genetic structure of the population (allelic frequencies, Hardy-Weinberg and linkage disequilibria).

We also clarify the connection between the general genetic model and some reduced models. By restricting the number of alleles at each locus to two and setting allelic frequencies to half, the general genetic model is reduced to the *F*_2 _model. This simplification reduces the partition of genetic variance enormously.

Another property for this *F*_2 _population is that, if there is no crossing-over interference, the three-locus linkage disequilibrium is expected to be zero regardlessly whether the loci are linked. Also the four-locus disequilibrium is reduced to the product of the two-locus disequilibria for the two non-adjacent locus pairs. If there is crossing-over interference, the three-locus linkage disequilibrium would be a good measure of the interference. As many QTL mapping experiments are performed in a *F*_2 _population, this reduced model is very relevant to QTL mapping analysis for the interpretation of genetic architecture in a *F*_2 _population. Another reduced model is the backcross model which is essentially a haploid model.

We give many details for a general two-allele model with epistasis and linkage disequilibrium. Research on QTL mapping analysis has been shifted in recent years from inbred line crosses to natural populations. With the availability of very dense SNP markers, it is now possible to use SNP for fine mapping of QTL in a natural population. Currently most QTL fine mapping studies are concentrated on candidate genes. It will be increasingly possible to have genome-wide SNP data for a sample of individuals from a natural population. The general two-allele model can be used as a framework to interpret and estimate the genome-wide genetic architecture for a quantitative trait in a natural population. The model can be extended to multiple alleles to take haplotypes into account if needed.

## Authors' contributions

TW conducted the initial derivation of models and partition of variance and drafted the initial manuscript. ZBZ extended the derivation of models and partition of variance, revised and finalized the manuscript. Both authors have read and approved the final manuscript. Comments and requests should be addressed to ZBZ or TW.

## Appendix

### A. Cockerham least squares estimates

In this appendix, we show that the regression coefficients (genetic effects) in model (7) are Cockerham least squares estimates under Hardy-Weinberg, linkage and genotypic equilibria. First, note that at each locus only one allele is present on a gamete. That is if an individual inherits an allele *A*_*i *_from a parental gamete, $z_{M_{i}}^{\left(1\right)}$ = 1 and the other $z_{M_{j}}^{\left(1\right)}$ = 0 for *j *≠ *i *Therefore, when *i *≠ *j*, we have *E*($z_{M_{i}}^{\left(1\right)}z_{M_{j}}^{\left(1\right)}$) = 0 or $E(x_{M_{i}}^{\left(1\right)}x_{M_{j}}^{\left(1\right)})=E(z_{M_{i}}^{\left(1\right)}z_{M_{j}}^{\left(1\right)})-E(z_{M_{i}}^{\left(1\right)})E(z_{M_{j}}^{\left(1\right)})=-p^{i}p^{j}$. Using these relationships, we can show the following for model (7).

• Additive effects: For *i *= 1, 2, ..., *n*_1_, we can show

$\begin{array}{l} E[(G-\mu )x_{M_{i}}^{\left(1\right)}]=E[(\sum_{i'}\alpha^{i'}x_{M_{i'}}^{\left(1\right)})x_{M_{i}}^{\left(1\right)}]=\alpha^{i}E(x_{M_{i}}^{(1)2})+\sum_{i'\neq i}\alpha^{i'}E(x_{M_{i}^{'}}^{\left(1\right)}x_{M_{i}}^{\left(1\right)})\\ =\alpha^{i}p^{i}(1-p^{i})+\sum_{i'\neq i}\alpha^{i'}(-p^{i'}p^{i})=\alpha^{i}p^{i}+(-p^{i})\sum_{i'}\alpha^{i'}p^{i'}=\alpha^{i}p^{i}, \end{array}$

On the other hand,

$\begin{array}{l} E[(G-\mu )x_{M_{i}}^{\left(1\right)}]=E(G\;x_{M_{i}}^{\left(1\right)})=E[G(z_{M_{i}}^{\left(1\right)}-p^{i})]=E(Gz_{M_{i}}^{\left(1\right)})-p^{i}E(G)\\ =E[E(Gz_{M_{i}}^{\left(1\right)}|z_{M_{i}}^{\left(1\right)})]-p^{i}G_{..}^{..}=p^{i}E(G|z_{M_{i}}^{\left(1\right)}=1)-p^{i}G_{..}^{..}=p^{i}(G_{..}^{i.}-G_{..}^{..}). \end{array}$

Therefore, $\alpha^{i}=G_{..}^{i.}-G_{..}^{..}$ for *i *= 1, 2, ..., *n*_1_. Similarly, we can show that $\alpha_{j}=G_{j.}^{..}-G_{..}^{..}$ for *j *= 1, 2, ..., *n*_1_, and $\beta^{k}=G_{..}^{.k}-G_{..}^{..}$, $\beta_{l}=G_{.l}^{..}-G_{..}^{..}$ for *k*, *l *= 1, 2, ..., *n*_2_.

• Dominance effects: For locus 1, we have

E[(G−μ)xMi(1)xFj(1)]=E[(∑i',j'δj'i'xMi'(1)xFj'(1))xMi(1)xFj(1)]=∑j'δj'iE(xMi(1)2)E(xFj(1)xFj'(1))+∑i'≠i∑j'δj'i'E(xMi(1)xMi'(1))E(xFj(1)xFj'(1))=pi(1−pi)∑j'δj'iE(xFj(1)xFj'(1))+∑i'≠i(−pipi')∑j'δj'i'E(xFj(1)xFj'(1))=pi∑j'δj'iE(xFj(1)xFj'(1))−∑i'pipi'∑j'δj'iE(xFj(1)xFj'(1))=pi(pjδji+∑j'δj'ipj')−pi∑j'E(xFj(1)xFj'(1))∑i'pi'δj'i'=pipjδji for i,j=1,2,…,n1.

On the other hand,

$\begin{array}{l} E[(G-\mu )x_{M_{i}}^{\left(1\right)}x_{F_{j}}^{\left(1\right)}]=E[G(z_{M_{i}}^{\left(1\right)}-p^{i})(z_{F_{j}}^{\left(1\right)}-p_{j})]\\ =E(Gz_{M_{i}}^{\left(1\right)}z_{F_{j}}^{\left(1\right)}-p^{i}E(Gz_{F_{j}}^{\left(1\right)})-p_{j}E(Gz_{M_{i}}^{\left(1\right)})+p^{i}p_{j}E(G)\\ =E[E(Gz_{M_{i}}^{\left(1\right)}z_{F_{j}}^{\left(1\right)}|z_{M_{i}}^{\left(1\right)},z_{F_{j}}^{\left(1\right)})]-p^{i}E[E(Gz_{F_{j}}^{\left(1\right)}|z_{F_{j}}^{\left(1\right)}])-p_{j}E[E(Gz_{M_{i}}^{\left(1\right)}|z_{M_{i}}^{\left(1\right)})]+p^{i}p_{j}G_{..}^{..}\\ =p^{i}p_{j}(G_{j.}^{i.}-G_{..}^{i.}-G_{j.}^{..}+G_{..}^{..}) \end{array}$

Therefore, $\delta_{j}^{i}=G_{j.}^{i.}-G_{..}^{i.}-G_{j.}^{..}+G_{..}^{..}$ for *i*, *j *= 1, 2, ..., *n*_1_. Similar results can be derived for other dominance terms at locus 2.

• Additive × additive effects: Note that

$\begin{array}{l} E[(G-\mu )x_{M_{i}}^{\left(1\right)}x_{M_{k}}^{\left(2\right)}]=\sum_{i^{\prime },k^{\prime }}(\alpha^{i^{\prime }}\beta^{k^{\prime }})E(x_{M_{i}}^{\left(1\right)}x_{M_{i^{\prime }}}^{\left(1\right)})E(x_{M_{k}}^{\left(2\right)}x_{M_{k^{\prime }}}^{\left(2\right)})\\ =\sum_{k^{'}}\left(\alpha^{i}\beta^{k^{\prime }})E(x_{M_{i}}^{(1)2})E(x_{M_{k}}^{\left(2\right)}x_{M_{k^{\prime }}}^{\left(2\right)}\right)+\sum_{i^{\prime }\neq i}\sum_{k^{\prime }}\left(\alpha^{i^{\prime }}\beta^{k^{\prime }}\right)(-p^{i}p^{i^{\prime }})E(x_{M_{k}}^{\left(2\right)}x_{M_{k^{\prime }}}^{\left(2\right)})\\ =p^{i}\sum_{k^{\prime }}\left(\alpha^{i}\beta^{k^{\prime }}\right)E(x_{M_{k}}^{\left(2\right)}x_{M_{k^{\prime }}}^{\left(2\right)})-p^{i}\sum_{i^{\prime }}\sum_{k^{\prime }}\left(\alpha^{i^{\prime }}\beta^{k^{\prime }}\right)p^{i^{\prime }}E(x_{M_{k}}^{\left(2\right)}x_{M_{k^{\prime }}}^{\left(2\right)})\\ =p^{i}q^{k}(\alpha^{i}\beta^{k^{\prime }}) \end{array}$

and

$E[(G-\mu )x_{M_{i}}^{\left(1\right)}x_{M_{k}}^{\left(2\right)}]=E(Gx_{M_{i}}^{\left(1\right)}x_{M_{k}}^{\left(2\right)})=E[G(z_{M_{i}}^{\left(1\right)}-p^{i})(z_{M_{k}}^{\left(2\right)}-q^{k})]=p^{i}q^{k}(G_{..}^{ik}-G_{..}^{i.}-G_{..}^{.k}+G_{..}^{..})$

We have $(\alpha^{i}\beta^{k})=G_{..}^{ik}-G_{..}^{i.}-G_{..}^{.k}+G_{..}^{..}$ for *i *= 1, 2, ..., *n*_1 _and *k *= 1, 2, ..., *n*_2_. Similar results can be derived for other additive by additive terms.

• Additive × dominance effects: Note that

$\begin{array}{l} E[(G-\mu )x_{M_{i}}^{\left(1\right)}x_{M_{k}}^{\left(2\right)}x_{F_{l}}^{\left(2\right)}]=E[x_{M_{i}}^{\left(1\right)}x_{M_{k}}^{\left(2\right)}x_{F_{l}}^{\left(2\right)}\sum_{i^{\prime },k^{\prime },l^{\prime }}(\alpha^{i^{\prime }}\gamma_{l^{\prime }}^{k^{\prime }})x_{M_{i^{\prime }}}^{\left(1\right)}x_{M_{k^{\prime }}}^{\left(2\right)}x_{F_{l^{\prime }}}^{\left(2\right)}]\\ =\sum_{k^{\prime },l^{\prime }}(\alpha^{i}\gamma_{l^{\prime }}^{k^{\prime }})E(x_{M_{i}}^{(1)2})E(x_{M_{k}}^{\left(2\right)}x_{M_{k^{\prime }}}^{\left(2\right)})E(x_{F_{l}}^{\left(2\right)}x_{F_{l^{\prime }}}^{\left(2\right)})=p^{i}\sum_{k^{\prime },l^{\prime }}(\alpha^{i}\gamma_{l^{\prime }}^{k^{\prime }})E(x_{M_{k}}^{\left(2\right)}x_{M_{k^{\prime }}}^{\left(2\right)})E(x_{F_{l}}^{\left(2\right)}x_{F_{l^{\prime }}}^{\left(2\right)})=p^{i}q^{k}q_{l}(\alpha^{i}\gamma_{l}^{k}) \end{array}$

and

$\begin{array}{l} E[(G-\mu )x_{M_{i}}^{\left(1\right)}x_{M_{k}}^{\left(2\right)}x_{F_{l}}^{\left(2\right)}]=E[G(z_{M_{i}}^{\left(1\right)}-p^{i})(z_{M_{k}}^{\left(2\right)}-q^{k})(z_{F_{l}}^{\left(2\right)}-q_{l})]\\ =p^{i}q^{k}q_{l}(G_{.l}^{ik}-G_{..}^{ik}-G_{.l}^{i.}-G_{.l}^{.k}+G_{..}^{i.}+G_{..}^{.k}+G_{.l}^{..}-G_{..}^{..}) \end{array}$

For *i *= 1, 2, ..., *n*_1 _and *k*, *l *= 1, 2, ..., *n*_2_, we have

$(\alpha^{i}\gamma_{l}^{k})=G_{.l}^{ik}-G_{..}^{ik}-G_{.l}^{i.}-G_{.l}^{.k}+G_{..}^{i.}+G_{..}^{.k}+G_{.l}^{..}-G_{..}^{..}$

Similar results can be derived for dominance by additive terms.

• Dominance × dominance effects: Note that

$\begin{array}{l} E[(G-\mu )x_{M_{i}}^{\left(1\right)}x_{F_{j}}^{\left(1\right)}x_{M_{k}}^{\left(2\right)}x_{F_{l}}^{\left(2\right)}]=E[x_{M_{i}}^{\left(1\right)}x_{F_{j}}^{\left(1\right)}x_{M_{k}}^{\left(2\right)}x_{F_{l}}^{\left(2\right)}\sum_{i^{\prime },j^{\prime },k^{\prime },l^{\prime }}\left(\delta_{j^{\prime }}^{i^{\prime }}\gamma_{l^{\prime }}^{k^{\prime }}\right)x_{M_{i^{\prime }}}^{\left(1\right)}x_{F_{j^{\prime }}}^{\left(1\right)}x_{M_{k^{\prime }}}^{\left(2\right)}x_{F_{l^{\prime }}}^{\left(2\right)}]\\ =\sum_{i^{\prime },j^{\prime }}E(x_{M_{i}}^{\left(1\right)}x_{M_{i^{\prime }}}^{\left(1\right)})E(x_{F_{j}}^{\left(1\right)}x_{F_{j^{\prime }}}^{\left(1\right)})\sum_{k^{\prime },l^{\prime }}\left(\delta_{j^{\prime }}^{i^{\prime }}\gamma_{l^{\prime }}^{k^{\prime }})E(x_{M_{k}}^{\left(2\right)}x_{M_{k^{\prime }}}^{\left(2\right)}\right)E(x_{F_{l}}^{\left(2\right)}x_{F_{l^{\prime }}}^{\left(2\right)})\\ =\sum_{i^{\prime },j^{\prime }}E(x_{M_{i}}^{\left(1\right)}x_{M_{i^{\prime }}}^{\left(1\right)})E(x_{F_{j}}^{\left(1\right)}x_{F_{j^{\prime }}}^{\left(1\right)})(\delta_{j^{\prime }}^{i^{\prime }}\gamma_{l}^{k})q^{k}q_{l}=(\delta_{j}^{i}\gamma_{l}^{k})p^{i}p_{j}q^{k}q_{l} \end{array}$

On the other hand

$\begin{array}{l} E[(G-\mu )x_{M_{i}}^{\left(1\right)}x_{F_{j}}^{\left(1\right)}x_{M_{k}}^{\left(2\right)}x_{F_{l}}^{\left(2\right)}]=E[G(z_{M_{i}}^{\left(1\right)}-p^{i})(x_{F_{j}}^{\left(1\right)}-p_{j})(z_{M_{k}}^{\left(2\right)}-q^{k})(z_{F_{l}}^{\left(2\right)}-q_{l})]\\ =p^{i}p_{j}q^{k}q_{l}(G_{jl}^{ik}-G_{j.}^{ik}-G_{.l}^{ik}-G_{jl}^{i.}-G_{jl}^{.k}+G_{..}^{ik}+G_{jl}^{..}\\ +G_{j.}^{i.}+G_{.l}^{.k}+G_{j.}^{.k}+G_{.l}^{i.}-G_{..}^{i.}-G_{..}^{.k}-G_{j.}^{..}-G_{.l}^{..}+G_{..}^{..}) \end{array}$

For *i*, *j *= 1, 2, ..., *n*_1 _and *k*, *l *= 1, 2, ..., *n*_2_, we have

$(\delta_{j}^{i}\gamma_{l}^{k})=G_{jl}^{ik}-G_{j.}^{ik}-G_{.l}^{ik}-G_{jl}^{i.}-G_{jl}^{.k}+G_{..}^{ik}+G_{jl}^{..}+G_{j.}^{i.}+G_{.l}^{.k}+G_{j.}^{.k}+G_{.l}^{i.}-G_{..}^{i.}-G_{..}^{.k}-G_{j.}^{..}-G_{.l}^{..}+G_{..}^{..}$

### B. Partition of genotypic variance in linkage equilibrium

Here we show that the genotypic variance *V*_*G *_of model (7) has the orthogonal partition (4) under Hardy-Weinberg, linkage and genotypic equilibria. First, note that the index variables $x_{M_{i}}^{\left(1\right)}$, $x_{F_{j}}^{\left(1\right)}$, $x_{M_{k}}^{\left(2\right)}$, $x_{F_{l}}^{\left(2\right)}$ have expectation zero. Second, the assumption of Hardy-Weinberg, linkage and genotypic equilibria mean that all alleles in different gametes and loci are independent so that, for example,

$Cov(x_{M_{i}}^{\left(r\right)},x_{F_{j}}^{\left(r\right)})=E(x_{M_{i}}^{\left(r\right)}x_{F_{j}}^{\left(r\right)})=E(x_{M_{i}}^{\left(r\right)})E(x_{F_{j}}^{\left(r\right)})=0$

Thus the additive and dominance effects within a locus are orthogonal to each other because

$Cov(x_{M_{k}}^{\left(r\right)},x_{M_{i}}^{\left(r\right)},x_{F_{j}}^{\left(r\right)})=E(x_{M_{k}}^{\left(r\right)}x_{M_{i}}^{\left(r\right)}x_{F_{j}}^{\left(r\right)})=E(x_{M_{k}}^{\left(r\right)})E(x_{M_{i}}^{\left(r\right)})E(x_{F_{j}}^{\left(r\right)})=0$

for any 1 ≤ *i*, *j*, *k *≤ *n*_1 _and locus *r *= 1 or 2. Similarly, the epistatic effects between loci are orthogonal to additive and dominance effects and also to other epistatic effects. Therefore, the total genotypic variance *V*_*G *_can be partitioned as

$V_{G}=V_{A_{1}}+V_{A_{2}}+V_{D_{1}}+V_{D_{2}}+V_{A_{1}A_{2}}+V_{A_{1}D_{2}}+V_{D_{1}A_{2}}+V_{D_{1}D_{2}}$

with each component analyzed below.

• The additive variance: For locus 1,

$\begin{array}{c} V_{A_{1}}=Var(\sum_{i=1}^{n_{1}}\alpha^{i}x_{M_{i}}^{\left(1\right)}+\sum_{j=1}^{n_{1}}\alpha_{j}x_{F_{j}}^{\left(1\right)})=Var(\sum_{i=1}^{n_{1}}\alpha^{i}x_{M_{i}}^{\left(1\right)})+Var(\sum_{j=1}^{n_{1}}\alpha_{j}x_{F_{j}}^{\left(1\right)})\\ =E[{\left(\sum_{i=1}^{n_{1}}\alpha^{i}x_{M_{i}}^{\left(1\right)}\right)}^{2}]+E[{\left(\sum_{j=1}^{n_{1}}\alpha_{j}x_{F_{j}}^{\left(1\right)}\right)}^{2}]\\ =E{\left[(\sum_{i=1}^{n_{1}}\alpha^{i}z_{M_{i}}^{\left(1\right)})-\sum_{i=1}^{n_{1}}\alpha^{i}p^{i}\right)}^{2}]+E[{\left(\sum_{j=1}^{n_{1}}\alpha_{j}x_{F_{j}}^{\left(1\right)}-\sum_{j=1}^{n_{1}}\alpha_{j}p_{j}\right)}^{2}]\\ =E[{\left(\sum_{i=1}^{n_{1}}\alpha^{i}z_{M_{i}}^{\left(1\right)}\right)}^{2}]+E[{\left(\sum_{j=1}^{n_{1}}\alpha_{j}z_{F_{j}}^{\left(1\right)}\right)}^{2}]\\ =\sum_{i=1}^{n_{1}}{\left(\alpha^{i}\right)}^{2}E[{\left(z_{M_{i}}^{\left(1\right)}\right)}^{2}]+\sum_{j=1}^{n_{1}}{\left(\alpha_{j}\right)}^{2}E[{\left(z_{F_{j}}^{\left(1\right)}\right)}^{2}]\\ =\sum_{i=1}^{n_{1}}{\left(\alpha^{i}\right)}^{2}p^{i}+\sum_{j=1}^{n_{1}}{\left(\alpha_{j}\right)}^{2}p_{j} \end{array}$

as $\sum_{i=1}^{n_{1}}\alpha^{i}p^{i}=0$ by the constrain condition (2). Similarly, for locus 2, we have $V_{A_{2}}=\sum_{k=1}^{n_{2}}{\left(\beta^{k}\right)}^{2}q^{k}+\sum_{j=1}^{n_{2}}{\left(\beta_{l}\right)}^{2}q_{l}$.

• The dominance variance: For locus 1,

$\begin{array}{c} V_{D_{1}}=Var(\sum_{i,j}\delta_{j}^{i}x_{M_{i}}^{\left(1\right)}x_{F_{j}}^{\left(1\right)})=E{\left[(\sum_{i,j}\delta_{j}^{i}(z_{M_{i}}^{\left(1\right)})-p^{i})(z_{F_{j}}^{\left(1\right)}-p_{j})\right)}^{2}]\\ =E[{\left(\sum_{i,j}\delta_{j}^{i}z_{M_{i}}^{\left(1\right)}z_{F_{j}}^{\left(1\right)}-\sum_{i,j}p_{j}\delta_{j}^{i}z_{M_{i}}^{\left(1\right)}-\sum_{i,j}p^{i}\delta_{j}^{i}z_{F_{j}}^{\left(1\right)}+\sum_{i,j}p^{i}p_{j}\delta_{j}^{i}\right)}^{2}]\\ =E[{\left(\sum_{i,j}\delta_{j}^{i}z_{M_{i}}^{\left(1\right)}z_{F_{j}}^{\left(1\right)}\right)}^{2}]=\sum_{i,j}{\left(\delta_{j}^{i}\right)}^{2}E(z_{M_{i}}^{(1)2})E(z_{F_{j}}^{(1)2})=\sum_{i,j}{\left(\delta_{j}^{i}\right)}^{2}p^{i}p_{j} \end{array}$

Similarly, for locus 2, we have $V_{D_{2}}=\sum_{k,l}{\left(\gamma_{l}^{k}\right)}^{2}q^{k}q_{l}$.

• The additive × additive variance:

$\begin{array}{c} V_{A_{1}A_{2}}=Var(\sum_{i,k}\left(\alpha^{i}\beta^{k})x_{M_{i}}^{\left(1\right)}x_{M_{k}}^{\left(2\right)}\right)+Var(\sum_{i,l}\left(\alpha^{i}\beta_{l})x_{M_{i}}^{\left(1\right)}x_{F_{l}}^{\left(2\right)}\right)\\ +Var(\sum_{j,k}\left(\alpha_{j}\beta^{k})x_{F_{j}}^{\left(1\right)}x_{M_{k}}^{\left(2\right)}\right)+Var(\sum_{j,l}\left(\alpha_{j}\beta_{l})x_{F_{j}}^{\left(1\right)}x_{F_{l}}^{\left(2\right)}\right)\\ =\sum_{i,k}p^{i}q^{k}{\left(\alpha^{i}\beta^{k}\right)}^{2}+\sum_{j,l}p_{j}q_{l}{\left(\alpha_{j}\beta_{l}\right)}^{2}+\sum_{i,l}p^{i}q_{l}{\left(\alpha^{i}\beta_{l}\right)}^{2}+\sum_{j,k}p_{j}q^{k}{\left(\alpha_{j}\beta^{k}\right)}^{2} \end{array}$

• The additive × dominance variance:

$\begin{array}{c} V_{A_{1}D_{2}}=Var(\sum_{i,k,l}\left(\alpha^{i}\gamma_{l}^{k}\right)x_{M_{i}}^{\left(1\right)}x_{M_{k}}^{\left(2\right)}x_{F_{l}}^{\left(2\right)})+Var(\sum_{j,k,l}\left(\alpha_{j}\gamma_{l}^{k}\right)x_{F_{j}}^{\left(1\right)}x_{M_{k}}^{\left(2\right)}x_{F_{l}}^{\left(2\right)})\\ =\sum_{i,k,l}p^{i}q^{k}q_{l}{\left(\alpha^{i}\gamma_{l}^{k}\right)}^{2}+\sum_{j,k,l}p_{j}q^{k}q_{l}{\left(\alpha_{j}\gamma_{l}^{k}\right)}^{2} \end{array}$

Similarly, the dominance × additive variance is $V_{D_{1}A_{2}}=\sum_{i,j,k}p^{i}p_{j}q^{k}{\left(\delta_{j}^{i}\beta^{k}\right)}^{2}+\sum_{i,j,l}p^{i}p_{j}q_{l}{\left(\delta_{j}^{i}\beta_{l}\right)}^{2}$.

• The dominance × dominance variance:

$\begin{array}{c} V_{D_{1}D_{2}}=Var(\sum_{i,j,k,l}(\delta_{j}^{i}\gamma_{l}^{k})x_{M_{i}}^{\left(1\right)}x_{F_{j}}^{\left(1\right)}x_{M_{k}}^{\left(2\right)}x_{F_{l}}^{\left(2\right)})\\ =E[{\left(\sum_{i,j,k,l}(\delta_{j}^{i}\gamma_{l}^{k})z_{M_{i}}^{\left(1\right)}z_{F_{j}}^{\left(1\right)}z_{M_{k}}^{\left(2\right)}z_{F_{l}}^{\left(2\right)}\right)}^{2}]=\sum_{i,j,k,l}p^{i}p_{j}q^{k}q_{l}{\left(\delta_{j}^{i}\gamma_{l}^{k}\right)}^{2} \end{array}$

### C. Partition of genotypic variance in linkage disequilibrium

We present the partition of the genotypic variance based on model (7) under the assumption of Hardy-Weinberg equilibrium but linkage disequilibrium.

• The additive variance:

$V_{A_{1}}=\sum_{i=1}^{n_{1}}p^{i}{\left(\alpha^{i}\right)}^{2}+\sum_{j=1}^{n_{1}}p_{j}{\left(\alpha_{j}\right)}^{2},\;V_{A_{2}}=\sum_{k=1}^{n_{2}}q^{k}{\left(\beta^{k}\right)}^{2}+\sum_{j=1}^{n_{2}}q_{l}{\left(\beta_{l}\right)}^{2}$

• The dominance variance:

$V_{D_{1}}=\sum_{i,j}{\left(\delta_{j}^{i}\right)}^{2}p^{i}p_{j},\;\;\;V_{D_{2}}=\sum_{k,l}{\left(\gamma_{l}^{k}\right)}^{2}q^{k}q_{l}$

• The additive × additive variance:

$\begin{array}{ll} V_{A_{1}A_{2}}= & \sum_{i,k}(p^{i}q^{k}+D_{..}^{ik}){\left(\alpha^{i}\beta^{k}\right)}^{2}-[\sum_{i,k}\left(\alpha^{i}\beta^{k}\right)D_{..}^{ik}{]}^{2}+\sum_{j,l}(p_{j}q_{l}+D_{jl}^{..}){\left(\alpha_{j}\beta_{l}\right)}^{2}\\ & -[\sum_{j,l}(\alpha_{j}\beta_{l})D_{jl}^{..}{]}^{2}+\sum_{i,l}p^{i}q_{l}{\left(\alpha^{i}\beta_{l}\right)}^{2}+\sum_{j,k}p_{j}q^{k}{\left(\alpha_{j}\beta^{k}\right)}^{2}+2\sum_{i,j,k,l}(\alpha^{i}\beta^{k})(\alpha_{j}\beta_{l})D_{jl}^{..}D_{..}^{ik}\\ & +2\sum_{i,j,k,l}(\alpha^{i}\beta_{l})(\alpha_{j}\beta^{k})D_{jl}^{..}D_{..}^{ik}+2(\sum_{i,k}\left(\alpha^{i}\beta^{k})D_{..}^{ik}\right)(\sum_{j,l}\left(\alpha_{j}\beta_{l})D_{jl}^{..}\right) \end{array}$

• The additive × dominance variance:

$V_{A_{1}D_{2}}=\sum_{i,k,l}q_{l}(p^{i}q^{k}+D_{..}^{ik}){\left(\alpha^{i}\gamma_{l}^{k}\right)}^{2}+\sum_{j,k,l}q^{k}(p_{j}q_{l}+D_{jl}^{..}){\left(\alpha_{j}\gamma_{l}^{k}\right)}^{2}+2\sum_{i,j,k,l}\left(\alpha^{i}\gamma_{l}^{k}\right)(\alpha_{j}\gamma_{l}^{k})D_{jl}^{..}D_{..}^{ik}$

• The dominance × additive variance:

$V_{D_{1}A_{2}}=\sum_{i,j,k}p_{j}(p^{i}q^{k}+D_{..}^{ik}){\left(\delta_{j}^{i}\beta^{k}\right)}^{2}+\sum_{i,j,l}p^{i}(p_{j}q_{l}+D_{jl}^{..}){\left(\delta_{j}^{i}\beta_{l}\right)}^{2}+2\sum_{i,j,k,l}\left(\delta_{j}^{i}\beta^{k}\right)(\delta_{j}^{i}\beta_{l})D_{jl}^{..}D_{..}^{ik}$

• The dominance × dominance variance:

$V_{D_{1}D_{2}}=\sum_{i,j,k,l}{\left(\delta_{j}^{i}\gamma_{l}^{k}\right)}^{2}(D_{..}^{ik}+p^{i}q^{k})(D_{jl}^{..}+p_{j}q_{l})$

• The covariances related to additive and dominance effects:

$\begin{array}{l} Cov(A_{1},A_{2})=\sum_{i,k}(\alpha^{i}\beta^{k})D_{..}^{ik}+\sum_{j,l}(\alpha_{j}\beta_{l})D_{jl}^{..}\\ Cov(D_{1},D_{2})=\sum_{i,j,k,l}\left(\delta_{j}^{i}\right)(\gamma_{l}^{k})D_{jl}^{..}D_{..}^{ik}\\ Cov(A_{1},D_{1})=Cov(A_{1},D_{2})=Cov(A_{2},D_{1})=Cov(A_{2},D_{2})=0 \end{array}$

• The covariances related to additive × additive effects:

$\begin{array}{l} Cov(A_{1},A_{1}A_{2})=\sum_{i,k}(\alpha^{i})(\alpha^{i}\beta^{k})D_{..}^{ik}+\sum_{j,l}(\alpha_{j})(\alpha_{j}\beta_{l})D_{jl}^{..}\\ Cov(A_{2},A_{1}A_{2})=\sum_{i,k}(\beta^{k})(\alpha^{i}\beta^{k})D_{..}^{ik}+\sum_{j,l}(\beta_{l})(\alpha_{j}\beta_{l})D_{jl}^{..}\\ Cov(D_{1},A_{1}A_{2})=\sum_{i,j,k}p_{j}(\delta_{j}^{i})(\alpha_{j}\beta^{k})D_{..}^{ik}+\sum_{i,j,l}p^{i}(\delta_{j}^{i})(\alpha^{i}\beta_{l})D_{jl}^{..}\\ Cov(D_{2},A_{1}A_{2})=\sum_{i,k,l}q_{l}(\alpha^{i}\beta_{l})(\gamma_{l}^{k})D_{..}^{ik}+\sum_{j,k,l}q^{k}(\alpha_{j}\beta^{k})(\gamma_{l}^{k})D_{jl}^{..} \end{array}$

• The covariances related to additive × dominance and dominance × additive effects:

$\begin{array}{ccc} Cov(A_{1},A_{1}D_{2}) & = & \sum_{i,j,k,l}[(\alpha^{i})(\alpha_{j}\gamma_{l}^{k})+(\alpha_{j})(\alpha^{i}\gamma_{l}^{k})]D_{jl}^{..}D_{..}^{ik}\\ Cov(A_{2},A_{1}D_{2}) & = & \sum_{i,k,l}q_{l}(\beta_{l})(\alpha^{i}\gamma_{l}^{k})D_{..}^{ik}+\sum_{j,k,l}q^{k}(\beta^{k})(\alpha_{j}\gamma_{l}^{k})D_{jl}^{..}\\ Cov(A_{1},D_{1}A_{2}) & = & \sum_{i,j,k}p_{j}(\alpha_{j})(\delta_{j}^{i}\beta^{k})D_{..}^{ik}+\sum_{i,j,l}p^{i}(\alpha^{i})(\delta_{j}^{i}\beta_{l})D_{jl}^{..}\\ Cov(A_{2},D_{1}A_{2}) & = & \sum_{i,j,k,l}[(\beta^{k})(\delta_{j}^{i}\beta_{l})+(\beta_{l})(\delta_{j}^{i}\beta^{k})]D_{jl}^{..}D_{..}^{ik}\\ Cov(D_{1},A_{1}D_{2}) & = & \sum_{i,j,k,l}(\delta_{j}^{i})[(\alpha^{i}\gamma_{l}^{k})+(\alpha_{j}\gamma_{l}^{k})]D_{jl}^{..}D_{..}^{ik}\\ Cov(D_{2},A_{1}D_{2}) & = & \sum_{i,k,l}q_{l}(\gamma_{l}^{k})(\alpha^{i}\gamma_{l}^{k})D_{..}^{ik}+\sum_{j,k,l}q^{k}(\gamma_{l}^{k})(\alpha_{j}\gamma_{l}^{k})D_{jl}^{..}\\ Cov(D_{1},D_{1}A_{2}) & = & \sum_{i,j,k}p_{j}(\delta_{j}^{i})(\delta_{j}^{i}\beta^{k})D_{..}^{ik}+\sum_{i,j,l}p^{i}(\delta_{j}^{i})(\delta_{j}^{i}\beta_{l})D_{jl}^{..}\\ Cov(D_{2},D_{1}A_{2}) & = & \sum_{i,j,k,l}(\gamma_{l}^{k})[(\delta_{j}^{i}\beta^{k})+(\delta_{j}^{i}\beta_{l})]D_{jl}^{..}D_{..}^{ik}\\ Cov(A_{1}A_{2},A_{1}D_{2}) & = & \sum_{i,k,l}q_{l}(\alpha^{i}\beta_{l})(\alpha^{i}\gamma_{l}^{k})D_{..}^{ik}+\sum_{j,k,l}q^{k}(\alpha_{j}\beta^{k})(\alpha_{j}\gamma_{l}^{k})D_{jl}^{..}\\ & & +\sum_{i,j,k,l}\left[(\alpha^{i}\beta^{k})(\alpha_{j}\gamma_{l}^{k}\right)+(\alpha^{i}\beta_{l})(\alpha_{j}\gamma_{l}^{k})+(\alpha_{j}\beta^{k})(\alpha^{i}\gamma_{l}^{k})+(\alpha_{j}\beta_{l})(\alpha^{i}\gamma_{l}^{k})]D_{jl}^{..}D_{..}^{ik}\\ \text{Cov}(A_{1},A_{2},D_{1}A_{2}) & = & \sum_{i,j,k}p_{j}(\alpha_{j}\beta^{k})(\delta_{j}^{i}\beta^{k})D_{..}^{ik}+\sum_{i,j,l}p^{i}(\alpha^{i}\beta_{l})(\delta_{j}^{i}\beta_{l})D_{jl}^{..}\\ & & +\sum_{i,j,k,l}[(\alpha^{i}\beta^{k})(\delta_{j}^{i}\beta l)+(\alpha_{j}\beta^{k})(\delta_{j}^{i}\beta_{l})+(\alpha^{i}\beta_{l})(\delta_{j}^{i}\beta^{k})+(\alpha_{j}\beta_{l})(\delta_{j}^{i}\beta^{k})]D_{jl}^{..}D_{..}^{ik}\\ Cov(A_{1}D_{2},D_{1}A_{2}) & = & \sum_{i,j,k,l}\left[(\alpha^{i}\gamma_{l}^{k}\right)(\delta_{j}^{i}\beta^{k})(D_{..}^{ik}+p^{i}q^{k})D_{jl}^{..}+(\alpha^{i}\gamma_{l}^{k})(\delta_{j}^{i}\beta_{l})D_{jl}^{..}D_{..}^{ik}\\ & & +(\alpha_{j}\gamma_{l}^{k})(\delta_{j}^{i}\beta^{k})D_{jl}^{..}D_{..}^{ik}+(\alpha_{j}\gamma_{l}^{k})(\delta_{j}^{i}\beta_{l})(D_{jl}^{..}+p_{j}q_{l})D_{..}^{ik}] \end{array}$

• The covariances related to dominance × dominance effects:

$\begin{array}{c} Cov(A_{1},D_{1}D_{2})=\sum_{i,j,k,l}(\delta_{j}^{i}\gamma_{l}^{k})(\alpha^{i}+\alpha_{j})D_{jl}^{..}D_{..}^{ik}\\ Cov(A_{2},D_{1}D_{2})=\sum_{i,j,k,l}(\delta_{j}^{i}\gamma_{l}^{k})(\beta^{k}+\beta_{l})D_{jl}^{..}D_{..}^{ik}\\ Cov(D_{1},D_{1}D_{2})=\sum_{i,j,k,l}(\delta_{j}^{i}\gamma_{l}^{k})(\delta_{j}^{i})D_{jl}^{..}D_{..}^{ik}\\ Cov(D_{2},D_{1}D_{2})=\sum_{i,j,k,l}(\delta_{j}^{i}\gamma_{l}^{k})(\gamma_{l}^{k})D_{jl}^{..}D_{..}^{ik}\\ Cov(A_{1}A_{2},D_{1}D_{2})=-\left(\sum_{i,k}(\alpha^{i}\beta^{k})D_{..}^{ik}+\sum_{j,l}(\alpha_{j}\beta_{l})D_{jl}^{..}\right)\left(\sum_{i,j,k,l}(\delta_{j}^{i}\gamma_{l}^{k})D_{jl}^{..}D_{..}^{ik}\right)+\sum_{i,j,k,l}\left(\delta_{j}^{i}\gamma_{l}^{k}\right)\{\left[(\alpha^{i}\beta^{k}\right)\\ +(\alpha^{i}\beta_{l})+(\alpha_{j}\beta^{k})+(\alpha_{j}\beta_{l})]D_{jl}^{..}D_{..}^{ik}+(\alpha^{i}\beta^{k})p^{i}q^{k}D_{jl}^{..}+(\alpha_{j}\beta_{l})p_{j}q_{l}D_{..}^{ik}\}\\ Cov(A_{1}D_{2},D_{1}D_{2})=\sum_{i,j,k,l}(\delta_{j}^{i}\gamma_{l}^{k})[(\alpha^{i}\gamma_{l}^{k})(D_{..}^{ik}+p^{i}q^{k})D_{jl}^{..}+(\alpha_{j}\gamma_{l}^{k})(D_{jl}^{..}+p_{j}q_{l})D_{..}^{ik}]\\ Cov(D_{1}A_{2},D_{1}D_{2})=\sum_{i,j,k,l}(\delta_{j}^{i}\gamma_{l}^{k})[(\delta_{j}^{i}\beta^{k})(D_{..}^{ik}+p^{i}q^{k})D_{jl}^{..}+(\delta_{j}^{i}\beta_{l})(D_{jl}^{..}+p_{j}q_{l})D_{..}^{ik}] \end{array}$

### D. Partition of genotypic variance in *F*_2 _population with linkage disequilibrium

Detail of each component of equation (19) for *F*_2 _population is presented here.

• The additive variance:

$V_{A}=\frac{1}{2}\sum_{r=1}^{m}a_{r}^{2}+2\sum_{r\neq s}a_{r}a_{s}D_{rs}$

• The dominance variance:

$V_{D}=\frac{1}{4}\sum_{r=1}^{m}d_{r}^{2}+4\sum_{r\neq s}d_{r}d_{s}D_{rs}^{2}$

• The additive × additive variance:

$\begin{array}{c} V_{AA}=\frac{1}{4}\sum_{r<s}{\left(aa\right)}_{rs}^{2}+\sum_{r\neq s\neq s^{\prime }}{\left(aa\right)}_{rs}{\left(aa\right)}_{rs^{\prime }}D_{ss^{\prime }}\\ +\frac{1}{2}\sum_{r\neq s\neq r^{\prime }\neq s^{\prime }}{\left(aa\right)}_{rs}{\left(aa\right)}_{r^{\prime }s^{\prime }}(D_{rr^{\prime }ss^{\prime }}+D_{rs^{\prime }}D_{r^{\prime }s}+D_{rr^{\prime }}D_{ss^{\prime }}-D_{rs}D_{r^{\prime }s^{\prime }}) \end{array}$

• The additive × dominance variance:

$\begin{array}{c} V_{AD}=\frac{1}{8}\sum_{r\neq s}{\left(ad\right)}_{rs}^{2}+\frac{1}{2}\sum_{r\neq s}{\left(ad\right)}_{rs}{\left(ad\right)}_{sr}D_{rs}+2\sum_{r\neq s\neq s^{\prime }}{\left(ad\right)}_{rs}[{\left(ad\right)}_{rs^{\prime }}D_{ss^{\prime }}^{2}+{\left(ad\right)}_{ss^{\prime }}D_{ss^{\prime }}D_{rs^{\prime }}\\ +{\left(ad\right)}_{s^{\prime }r}D_{rs}D_{ss^{\prime }}+\frac{1}{4}{\left(ad\right)}_{s^{\prime }s}D_{rs^{\prime }}]+8\sum_{r\neq s\neq r^{\prime }\neq s^{\prime }}{\left(ad\right)}_{rs}{\left(ad\right)}_{r^{\prime }s^{\prime }}D_{rsr^{\prime }s^{\prime }}D_{ss^{\prime }} \end{array}$

• The dominance × dominance variance:

$\begin{array}{l} V_{DD}=\sum_{r<s}{\left(dd\right)}_{rs}^{2}(\frac{1}{16}-16D_{rs}^{4})+\sum_{r\neq s\neq s'}{\left(dd\right)}_{rs}{\left(dd\right)}_{rs'}(D_{ss'}^{2}-16D_{rs}^{2}D_{rs'}^{2})\\ +4\sum_{r\neq s\neq r'\neq s'}{\left(dd\right)}_{rs}{\left(dd\right)}_{r's'}(D_{rsr's'}^{2}-D_{rs}^{2}D_{r's'}^{2}) \end{array}$

• The covariances: Cov(*A*, *D*) = Cov(*A*, *AA*) = Cov(*A*, *DD*) = Cov(*D*, *AD*) = Cov(*D*, *DD*) = Cov(*AA*, *AD*) = Cov(*AD*, *DD*) = 0, and

$\begin{array}{c} \text{Cov}(A,AD)=-\sum_{r\neq s}a_{r}D_{rs}\left[4{\left(ad\right)}_{rs}D_{rs}+{\left(ad\right)}_{sr}\right]-4\sum_{r\neq s\neq r'}a_{r'}{\left(ad\right)}_{rs}D_{rs}D_{r's}\\ \text{Cov}(D,AA)=-\sum_{r<s}(d_{r}+d_{s}){\left(aa\right)}_{rs}D_{rs}-4\sum_{r\neq s\neq r'}d_{r'}{\left(aa\right)}_{rs}D_{rr'}D_{r's}\\ \text{Cov}(AA,DD)=\sum_{r<s}{\left(aa\right)}_{rs}{\left(dd\right)}_{rs}(\frac{1}{2}D_{rs}-8D_{rs}^{3})+\sum_{r\neq s\neq s'}{\left(aa\right)}_{rs}{\left(dd\right)}_{rs'}(2D_{rs'}D_{ss'}-8D_{rs}D_{rs'}^{2})\\ +2\sum_{r\neq s\neq r'\neq s'}{\left(aa\right)}_{rs}{\left(dd\right)}_{r's'}(D_{rsr's'}D_{r's'}-D_{rs}D_{r's'}^{2}) \end{array}$

where (*aa*)_*sr *_= (*aa*)_*rs *_and (*dd*)_*sr *_= (*dd*)_*rs *_for *r *<*s*.

In this presentation, we utilized the assumption of no crossing-over interference which results in the third order linkage disequilibrium of three loci being zero, *i.e*. *D*_*rss*' _= 0. It may be instructive to show this result.

$\begin{array}{c} D_{rst}=E(x_{r}x_{s}x_{t})=E((z_{r}-p_{r})(z_{s}-p_{s})(z_{t}-p_{t}))\\ =E(z_{r}z_{s}z_{t}-p_{r}z_{s}z_{t}-z_{r}p_{s}z_{t}-z_{r}z_{s}p_{t}+p_{r}p_{s}z_{t}+p_{r}z_{s}p_{t}+z_{r}p_{s}p_{t}-p_{r}p_{s}p_{t})\\ =E(z_{r}z_{s}z_{t}-\frac{1}{2}z_{s}z_{t}-\frac{1}{2}z_{r}z_{t}-\frac{1}{2}z_{r}z_{s}+\frac{1}{4}) \end{array}$

Let *r*_1 _be the recombination frequency between loci *r *and *s*, *r*_2 _be that between *s *and *t*, and *r*_12 _be that between *r *and *t*. Under the assumption of no crossing-over interference, for loci *r*, *s*, *t *in this order, we have *E*(*z*_*r*_*z*_*s*_*z*_*t*_) = $\frac{1}{2}$(1 - *r*_1_)(1 - *r*_2_), *E*(*z*_*r*_*z*_*s*_) = $\frac{1}{2}$(1 - *r*_1_), *E*(*z*_*s*_*z*_*t*_) = $\frac{1}{2}$(1 - *r*_2_), and *E*(*z*_*r*_*z*_*t*_) = $\frac{1}{2}$(1 - *r*_12_). Since the assumption of no crossing-over interferene implies *r*_12 _= *r*_1 _+ *r*_2 _- 2*r*_1_*r*_2_, thus *D*_*rst *_= 0.

### E. Partition of genotypic variance for the general two-allele model with linkage disequilibrium

Detail of each component of equation (19) for the general two-allele model is presented here.

• The additive variance:

$V_{A}=2\sum_{r=1}^{m}p_{r}(1-p_{r})a_{r}^{2}+2\sum_{r\neq s}a_{r}a_{s}D_{rs}$

• The dominance variance:

$V_{D}=\sum_{r=1}^{m}p_{r}^{2}{\left(1-p_{r}\right)}^{2}d_{r}^{2}+\sum_{r\neq s}d_{r}d_{s}D_{rs}^{2}$

• The additive × additive variance:

$\begin{array}{c} V_{AA}=2\sum_{r<s}{\left(aa\right)}_{rs}^{2}\left[\left(1-2p_{r})(1-2p_{s})D_{rs}+2p_{r}(1-p_{r})p_{s}(1-p_{s}\right)\right]\\ +2\sum_{r\neq s\neq s'}{\left(aa\right)}_{rs}{\left(aa\right)}_{rs'}\left[(1-2p_{r})D_{rss'}+2p_{r}(1-p_{r})D_{ss'}\right]\\ +\frac{1}{2}\sum_{r\neq s\neq r'\neq s'}{\left(aa\right)}_{rs}{\left(aa\right)}_{r's'}(D_{rr'ss'}+D_{rs'}D_{r's}+D_{rr'}D_{ss'}-D_{rs}D_{r's'}) \end{array}$

• The additive × dominance variance:

$\begin{array}{l} V_{AD}=2\sum_{r\neq s}{\left(ad\right)}_{rs}^{2}\left[{\left(1-2p_{s}\right)}^{2}D_{rs}^{2}+(1-2p_{r})(1-2p_{s})p_{s}(1-p_{s})D_{rs}+p_{r}(1-p_{r})p_{s}^{2}{\left(1-p_{s}\right)}^{2}\right]\\ +2\sum_{r\neq s}{\left(ad\right)}_{rs}{\left(ad\right)}_{sr}D_{rs}\left[2(1-2p_{r})(1-2p_{s})D_{rs}+p_{r}(1-p_{r})p_{s}(1-p_{s})\right]\\ +2\sum_{r\neq s\neq s'}{\left(ad\right)}_{rs}\{{\left(ad\right)}_{rs'}\left[D_{rss'}^{2}+(1-2p_{r})D_{rss'}D_{ss'}+p_{r}(1-p_{r})D_{ss'}^{2}\right]\\ +{\left(ad\right)}_{ss'}\left[2(1-2p_{s})D_{rss'}D_{ss'}+p_{s}(1-p_{s})D_{ss'}D_{rs'}\right]\\ +{\left(ad\right)}_{s'r}\left[2(1-2p_{r})D_{rss'}D_{rs}+p_{r}(1-p_{r})D_{rs}D_{ss'}\right]\\ +{\left(ad\right)}_{s's}\left[{\left(1-2p_{s}\right)}^{2}D_{rs}D_{ss'}+(1-2p_{s})p_{s}(1-p_{s})D_{rss'}+p_{s}^{2}{\left(1-p_{s}\right)}^{2}D_{rs'}\right]\}\\ +2\sum_{r\neq s\neq r'\neq s'}{\left(ad\right)}_{rs}{\left(ad\right)}_{r's'}(D_{rsr's'}D_{ss'}+D_{rss'}D_{sr's'}) \end{array}$

• The dominance × dominance variance:

$\begin{array}{lll} V_{DD} & = & \sum_{r<s}{\left(dd\right)}_{rs}^{2}\left\{{\left[\left(1-2p_{r})(1-2p_{s})D_{rs}+p_{r}(1-p_{r})p_{s}(1-p_{s}\right)\right]}^{2}-D_{rs}^{4}\right\}\\ & & +\sum_{r\neq s\neq s'}{\left(dd\right)}_{rs}{\left(dd\right)}_{rs'}\left\{{\left[(1-2p_{r})D_{rss'}+p_{r}(1-p_{r})D_{ss'}\right]}^{2}-D_{rs}^{2}D_{rs'}^{2}\right\}\\ & & +\frac{1}{4}\sum_{r\neq s\neq r'\neq s'}{\left(dd\right)}_{rs}{\left(dd\right)}_{r's'}(D_{rsr's'}^{2}-D_{rs}^{2}D_{r's'}^{2}) \end{array}$

• The covariances:

$\begin{array}{lll} \text{Cov}(A,D) & = & 0\\ \text{Cov}(A,AA) & = & 2\sum_{r<s}{\left(aa\right)}_{rs}D_{rs}\left[(1-2p_{r})a_{r}+(1-2p_{s})a_{s}\right]+\sum_{r\neq s\neq r'}a_{r'}{\left(aa\right)}_{rs}D_{r'rs}\\ \text{Cov}(A,AD) & = & 2\sum_{r\neq s}a_{r}D_{rs}\left[{\left(ad\right)}_{rs}D_{rs}+p_{r}(1-p_{r}){\left(ad\right)}_{sr}\right]+2\sum_{r\neq s\neq r'}a_{r'}{\left(ad\right)}_{rs}D_{rs}D_{r's}\\ \text{Cov}(A,DD) & = & 2\sum_{r<s}{\left(dd\right)}_{rs}D_{rs}^{2}\left[(1-2p_{r})a_{r}+(1-2p_{s})a_{s}\right]+2\sum_{r\neq s\neq r'}a_{r'}{\left(dd\right)}_{rs}D_{rsr'}D_{rs}\\ \text{Cov}(D,AA) & = & 2\sum_{r<s}{\left(aa\right)}_{rs}D_{rs}\left[p_{r}(1-p_{r})d_{r}+p_{s}(1-p_{s})d_{s}\right]+2\sum_{r\neq s\neq r'}d_{r'}{\left(aa\right)}_{rs}D_{rr'}D_{r's}\\ \text{Cov}(D,AD) & = & 2\sum_{r\neq s}d_{r}D_{rs}(1-2p_{r})\left[D_{rs}{\left(ad\right)}_{rs}+p_{r}(1-p_{r}){\left(ad\right)}_{sr}\right]+2\sum_{r\neq s\neq r'}d_{r'}{\left(ad\right)}_{rs}D_{r'rs}D_{r's}\\ Cov(D,DD) & = & \sum_{r<s}{\left(dd\right)}_{rs}D_{rs}^{2}\left[{\left(1-2p_{r}\right)}^{2}d_{r}+{\left(1-2p_{s}\right)}^{2}d_{s}\right]+2\sum_{r\neq s\neq r'}d_{r'}{\left(dd\right)}_{rs}D_{r'rs}^{2}\\ \text{Cov}(AA,AD) & = & 2\sum_{r<s}{\left(aa\right)}_{rs}D_{rs}\{{\left(ad\right)}_{rs}\left[(1-2p_{r})p_{s}(1-p_{s})+2(1-2p_{s})D_{rs}\right]\\ & & +{\left(ad\right)}_{sr}\left[(1-2p_{s})p_{r}(1-p_{r})+2(1-2p_{r})D_{rs}\right]\}\\ & & +2\sum_{r\neq s\neq s'}{\left(aa\right)}_{rs}\{{\left(ad\right)}_{ss'}\left[(1-2p_{s})D_{ss'}D_{rs'}+2D_{rss'}D_{ss'}\right]\\ & & +{\left(ad\right)}_{s's}\left[2(1-2p_{s})D_{rs}D_{ss'}+p_{s}(1-p_{s})D_{rss'}\right]\}\\ & & +\sum_{r\neq s\neq r'\neq s'}{\left(aa\right)}_{rs}{\left(ad\right)}_{r's'}(D_{rss'}D_{r's}+D_{rr's'}D_{ss'}+D_{r'ss'}D_{rs'})\\ \text{Cov}(AA,DD) & = & 2\sum_{r<s}{\left(aa\right)}_{rs}{\left(dd\right)}_{rs}\left[2(1-2p_{r})(1-2p_{s})D_{rs}^{2}+p_{r}(1-p_{r})p_{s}(1-p_{s})D_{rs}-D_{rs}^{3}\right]\\ & & +2\sum_{r\neq s\neq s'}{\left(aa\right)}_{rs}{\left(dd\right)}_{rs'}\left[2(1-2p_{r})D_{rss'}D_{rs'}+p_{r}(1-p_{r})D_{rs'}D_{ss'}-D_{rs}D_{rs'}^{2}\right]\\ & & +\frac{1}{2}\sum_{r\neq s\neq r'\neq s'}{\left(aa\right)}_{rs}{\left(dd\right)}_{r's'}(D_{rsr's'}D_{r's'}+D_{rr's'}D_{sr's'}-D_{rs}D_{r's'}^{2})\\ \text{Cov}(AD,DD) & = & 2\sum_{r\neq s}{\left(ad\right)}_{rs}{\left(dd\right)}_{rs}D_{rs}(1-2p_{s})\left[\left(1-2p_{r})(1-2p_{s})D_{rs}+p_{r}(1-p_{r})p_{s}(1-p_{s}\right)\right]\\ & & +2\sum_{r\neq s\neq s'}{\left(ad\right)}_{rs}\{{\left(dd\right)}_{rs'}D_{rss'}\left[(1-2p_{r})D_{rss'}+p_{r}(1-p_{r})D_{ss'}\right]\\ & & +{\left(dd\right)}_{ss'}D_{ss'}(1-2p_{s})\left[(1-2p_{s})D_{rss'}+p_{s}(1-p_{s})D_{rs'}\right]\}\\ & & +\sum_{r\neq s\neq r'\neq s'}{\left(ad\right)}_{rs}{\left(dd\right)}_{r's'}D_{rss'}D_{rsr's'} \end{array}$

where (*aa*)_*sr *_= (*aa*)_*rs *_and (*dd*)_*sr *_= (*dd*)_*rs *_for *r *<*s*.

The result in Appendix D for the *F*_2 _model with *p*_*r *_= 1/2 for *r *= 1, ..., *m *and also assuming *D*_*rss*' _= 0 is a special case of the results presented here. There is a difference, by a factor -2, on the specification of *v *variable for dominance effect for the *F*_2 _model and the general two-allele model, which carries over to the comparison of results in Appendix D and E.
